# Extracellular vesicle formation in *Lactococcus lactis* is stimulated by prophage‐encoded holin–lysin system

**DOI:** 10.1111/1751-7915.13972

**Published:** 2022-03-01

**Authors:** Yue Liu, Marcel H. Tempelaars, Sjef Boeren, Svetlana Alexeeva, Eddy J. Smid, Tjakko Abee

**Affiliations:** ^1^ Food Microbiology Wageningen University and Research P.O. Box 17 Wageningen 6700 AA the Netherlands; ^2^ Laboratory of Biochemistry Wageningen University and Research Wageningen the Netherlands

## Abstract

Gram‐positive bacterial extracellular membrane vesicles (EVs) have been drawing more attention in recent years. However, mechanistic insights are still lacking on how EVs are released through the cell walls in Gram‐positive bacteria. In this study, we characterized underlying mechanisms of EV production and provide evidence for a role of prophage activation in EV release using the Gram‐positive bacterium *Lactococcus lactis* as a model. By applying a standard EV isolation procedure, we observed the presence of EVs in the culture supernatant of a lysogenic *L*. *lactis* strain FM‐YL11, for which the prophage‐inducing condition led to an over 10‐fold increase in EV production in comparison with the non‐inducing condition. In contrast, the prophage‐encoded holin–lysin knockout mutant YL11ΔHLH and the prophage‐cured mutant FM‐YL12 produced constantly low levels of EVs. Under the prophage‐inducing condition, FM‐YL11 did not show massive cell lysis. Defective phage particles were found to be released in and associated with holin–lysin‐induced EVs from FM‐YL11, as demonstrated by transmission electron microscopic images, flow cytometry and proteomics analysis. Findings from this study further generalized the EV‐producing phenotype to Gram‐positive *L. lactis*, and provide additional insights into the EV production mechanism involving prophage‐encoded holin–lysin system. The knowledge on bacterial EV production can be applied to all Gram‐positive bacteria and other lactic acid bacteria with important roles in fermentations and probiotic formulations, to enable desired release and delivery of cellular components with nutritional values or probiotic effects.

## Graphical abstract

In this study we established that the prophage‐encoded holin and lysin stimulate extracellular vesicle (EV) production in *L. lactis*. The prophage holin‐lysin induced EVs are produced by gradual extrusion of the cytoplasmic membrane without immediate cell lysis, and may include phage particles as the cargos.

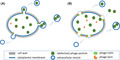

## Introduction

Cells from all domains of life produce extracellular membrane vesicles (EVs or MVs). These membrane‐enclosed, nano‐sized vesicles have been found to carry a variety of biomolecules and demonstrate various physiological and ecological functions (György *et al*., 2011; Gill *et al*., 2019). In the domain of bacteria, observations and studies of EVs started to appear several decades ago with the Gram‐negative bacteria, while studies of Gram‐positive bacterial EVs are catching up in recent years (Brown *et al*., 2015; Kim *et al*., 2015; Toyofuku *et al*., 2018).

EV‐producing Gram‐positive bacterial species have been revealed gradually, including a wide range of pathogens, probiotics and fermentation starters (Liu *et al*., 2018; Bose *et al*., 2020; Briaud and Carroll, 2020). Nucleic acids, viral particles, enzymes, receptors and many other effector molecules have been found to be the cargos of Gram‐positive EVs, associating EVs with roles in bacterial survival and competition in a microbial community, as well as in microbe–host interactions.

The delay in discovery of Gram‐positive bacterial EV production was largely due to the historical presumption that the cell wall of Gram‐positive bacteria, consisting of a thick peptidoglycan layer, would act as a strong physical barrier preventing the release of EVs generated from the cell membrane (Brown *et al*., 2015). Hence, the mechanism of Gram‐positive EV production has not been fully elucidated. A limited number of studies examining the production mechanism of Gram‐positive EVs so far contributed to theories on two steps: (i) budding of the cytoplasmic membrane and (ii) passage through the cell wall. Driving forces like turgor pressure and factors improving membrane fluidity were shown to facilitate the formation of membrane vesicles and cell‐wall modifications leading to compromised integrity were shown to be the key of EV release (Briaud and Carroll, 2020). To explain the latter, factors such as antibiotics that reduce the peptidoglycan cross‐linking, and cell‐wall degrading enzymes like autolysins and prophage‐derived endolysins, have been demonstrated to promote EV release in *Staphylococcus aureus* and *Bacillus subtilis* (Toyofuku *et al*., 2017; Andreoni *et al*., 2019).

Lactic acid bacteria (LAB) are an important group of Gram‐positive bacteria, with many species recognized for their probiotic effects as well as key players in food fermentation (Pasolli *et al*., 2020). EV production has been described for a number of LAB species, including *Lactiplantibacillus plantarum* [previously referred to as *Lactobacillus plantarum* (Zheng *et al*., 2020)] (Li *et al*., 2017) and *Lacticaseibacillus casei* [previously referred to as *Lactobacillus casei* (Zheng *et al*., 2020)] (Domínguez Rubio *et al*., 2017) and *Lactococcus lactis* (Liu *et al*., 2022).

*Lactococcus lactis* is extensively used for the production of fermented dairy products (Cavanagh *et al*., 2015). In the cases of artisanal cheese production, undefined complex starter cultures containing *L. lactis* strains are commonly used. Such complex cultures are usually shaped by historical use through sequential propagation of starters that lead to successful fermentation (back‐slopping), which eventually delivered a stable and robust microbial community (Erkus *et al*., 2013; Smid *et al*., 2014). Complex bacterial communities like undefined starter cultures often show diverse interactions with coexisting microorganisms and viruses, resulting in co‐evolution between, for instance, bacteriophages (phages) and bacteria and between different bacterial species and strains. Previous studies of an undefined complex cheese starter culture (named Ur) revealed many intriguing properties of members in such a microbial community: in this stable, robust culture, most *L. lactis* strains appeared to be lysogenic and to release defective tailless phage particles continuously in a chronic, non‐lytic manner, where the phage particles were enclosed in a lipid membrane layer (Alexeeva *et al*., 2018, 2021; Liu *et al*., 2022).

Given the abundance of prophages and intriguing phage–bacteria interactions of lysogenic *L. lactis* strains resident in complex dairy cultures (Alexeeva *et al*., 2018, 2021; Liu *et al*., 2022), as well as earlier findings on the prophage roles in Gram‐positive bacterial EV release (Toyofuku *et al*., 2017; Andreoni *et al*., 2019), we decided to investigate the role of prophages in EV release in *L. lactis*.

We isolated a *L. lactis* ssp. *cremoris* strain FM‐YL11 from an artisanal cheese (typically made with complex starter cultures), which shows similar growth behaviour as the previously described Ur culture strain TIFN1 under prophage‐inducing conditions (Alexeeva *et al*., 2018; Liu *et al*., 2022). Whole‐genome sequencing also revealed a prophage sequence in FM‐YL11 that is identical to proPhi1 and proPhi5 (Alexeeva *et al*., 2021) harboured by strains TIFN1 and TIFN5 in Ur, confirming that this strain is an ideal model to study the role of prophage in EV release. We demonstrated that *L. lactis* strain FM‐YL11 produces EVs, provided evidence for a role of phage holin–lysin in EV release and characterized the cargos and compositions of the EVs.

## Experimental procedures

### Strains and conditions

*Lactococcus lactis* ssp. *cremoris* strain FM‐YL11 is an isolate from artisanal cheese, and strain FM‐YL12 is a prophage‐cured derivative from strain FM‐YL11 obtained in the same manner as described by Alexeeva *et al*. (2018). Strains wild‐type FM‐YL11, prophage‐cured derivative FM‐YL12 and holin–lysin knockout mutant FM‐YL11ΔHLH were cultivated in M17 medium (Difco, BD Biosciences) supplemented with 0.5% (w/v) lactose (LM17) and statically incubated at 30°C, unless specified differently. The strain with gene complementation containing pNisA‐HLH and the control strain containing pNisA were cultivated in LM17 media supplemented with 3 µg ml^‐1^ erythromycin.

*Escherichia coli* strains used as plasmid hosts in this study were cultivated in LB broth (BD Difco) supplemented with 150 µg ml^‐1^ erythromycin, in Erlenmeyer flasks shaken at 120 rpm at 37°C.

### Genome sequencing

The original strain *L. lactis* FM‐YL11 and its prophage‐cured derivative strain FM‐YL12 were subjected to genome sequencing. The genomic DNA was isolated from 1 ml of overnight culture (cultivated in GM17 media) by using the DNeasy Blood & Tissue Kit (Qiagen, Hilden, Germany) according to the manufacture’s instruction. DNA was sequenced using the PacBio RSII technology and sequences were assembled by Hierarchical Genome Assembly Process (HGAP, v.3) (GATC Biotech, Konstanz, Germany). The genome coverages for strain FM‐YL11 and FM‐YL12 were 382x and 257x respectively.

The genome sequences of strain FM‐YL11 and FM‐YL12 can be accessed in GenBank under accession numbers CP071729 and CP071728 respectively. The genome annotation was performed using the NCBI Prokaryotic Genome Annotation Pipeline (PGAP).

### Mutant construction

#### Plasmid construction

Gene knockout in *L. lactis* FM‐YL11 was performed by homologous recombination. Based on 100% sequence identity of prophage sequence in FM‐YL11 to proPhi1 (Alexeeva *et al*., 2021), bases 1990275–1990499 of FM‐YL11 were annotated as a prophage holin gene, while bases 1986322–1987874 were annotated as a second holin next to a lysin encoding gene. All three genes were the targets for gene knockout.

To construct the plasmids for gene knockout, 600–800 bp upstream and downstream regions of the single holin gene as well as the holin–lysin genes were amplified by PCR, during which the restriction sites were introduced using primers listed in Table S1. Phusion High‐fidelity PCR kit (Thermo Fisher Scientific, Waltham, MA, USA) was used according to manufacturer’s instruction. The upstream and downstream homologous regions of the single holin gene and the holin–lysin gene cluster were inserted in plasmid pG^+^host9 (Maguin *et al*., 1992) by restriction digestion and ligation following enzyme product manuals from Thermo Fisher Scientific. In brief, restriction site PstI was used to connect the upstream and downstream homologous regions of the single holin gene, and the XhoI and EagI restriction sites were employed to insert the two homologous regions into pG^+^host9, yielding pYL006. Restriction site HindIII was used to connect the upstream and downstream homologous regions of the holin–lysin cluster, and the XhoI and PstI sites were employed to insert the two homologous regions into pG^+^host9, yielding pYL002.

For gene complementation, the coding regions of the holin–lysin cluster and the single holin gene were amplified by PCR using primers listed in Table S2. The two PCR products were inserted into the backbone (the 8515 bp XbaI ‐ PstI fragment) of plasmid pMSP3545 [gift from Gary Dunny (Bryan *et al*., 2000), Addgene plasmid # 46888]. NEBuilder HiFi DNA assembly cloning kit (New England Biolabs, Ipswich, MA, USA) was employed to assemble the three DNA fragments in one step according to the manufacturer’s instruction. This delivered plasmid pNisA‐HLH, in which the holin–lysin cluster as well as the single holin gene were inserted under the control of a nisin‐inducible promoter coming in pMSP3545.

#### Transformation

For construction of the plasmids for gene knockout, *E. coli* strain EC1000 (Leenhouts *et al*., 1998) was used for cloning and plasmid propagation. Treatment to obtain competent cells and heat‐shock transformation of EC1000 was performed as described by Chang *et al*. (2017) with the following modifications: prior to the two washing steps with 0.1 M CaCl_2_, the cells were washed with 1x volume of 0.1 M MgCl_2_. The heat‐shock at 42°C was for 90 s, followed by immediate addition of LB broth and incubation at 37°C statically for 1 h before plating on selection plates.

For constructing the plasmid for gene complementation, assembled plasmids were used to transform *E. coli* competent cells Mix & Go Zymo 5α (Zymo research, Irvine, CA, USA) according to manufacturer’s instructions.

Plasmids pYL002 and pYL006 were introduced into *L. lactis* FM‐YL11 to knock out the holin–lysin cluster and the single holin successively. Transformation of strain FM‐YL11 with plasmids pYL002 and pYL006 was performed as described previously (Alexeeva *et al*., 2018), except that transformed cells were selected and incubated at 28°C, which was the temperature that allows the replication of pG^+^host9‐derived plasmids (containing thermosensitive replication origin). Then, the plasmid integration and backbone elimination steps were performed as described by Liu *et al*. (2022), with lactose as the carbon source throughout. The only change was that the mutants propagated in SA medium were streaked on SA agar plates without 5‐fluoroorotate, and the selection of the correct knockout mutants was performed by colony screening using PCR.

Plasmid pNisA‐HLH for gene complementation was used to transform the knockout mutant FM‐YL11ΔHLH, exactly as described previously (Alexeeva *et al*., 2018). The empty vector pMSP3545, renamed as pNisA, was used to transform strain FM‐YL11ΔHLH to obtain a negative control strain for gene complementation.

### EV production and isolation

For EV production in strains FM‐YL11, FM‐YL12 and FM‐YL11ΔHLH, an overnight culture of *L. lactis* in LM17 was diluted to OD_600_ of 0.2 (light path 1 cm) in fresh LM17 medium and incubated for 1 h at 30⁰C. Then, 1 µg ml^‐1^ mitomycin C was added, which was referred to as the ‘prophage inducing condition’; where no mitomycin C was added, we refer to the ‘non‐inducing condition’. The OD_600_ of cultures was monitored every 30 min. After 6 h of treatment at prophage‐inducing condition or non‐inducing condition, EVs were harvested from the culture supernatant.

For EV production in strains with gene complementation (FM‐YL11ΔHLH containing pNisA‐HLH and control strain containing pNisA), 1 ml of overnight culture was diluted with 50 ml of fresh LM17 media supplemented with 3 µg ml^‐1^ erythromycin, and 10 ng ml^‐1^ nisin (Sigma‐Aldrich, Diegem, Belgium) was added. After 20 h of incubation, EVs were harvested from the culture supernatant.

To harvest EVs, the cell culture was centrifuged at 5000 *g* for 15 min at 4⁰C (low spin), and the supernatant was collected. The pH value of the supernatant was adjusted to 7.0 with 1 N NaOH. The supernatant was filtered through a Sartorius minisart polyethersulfone (PES) membrane filter with a pore size of 0.45 µm (Sartorius, Goettingen,Germany). The filtered supernatant was added to Beckman Coulter^®^ (Palo Alto, CA, USA) centrifuge tubes and centrifuged at 160 000 *g* for 1 h in a Beckman L60 Ultracentrifuge (high spin). The supernatant was discarded and the pellets were then re‐suspended in phosphate‐buffered saline (PBS) and aliquots stored at −80°C until further use. Repeated freezing and thawing were avoided by using new aliquots in the different experiments.

### EV quantification

Fifty millilitres of PBS with EVs in suspension was mixed with 50 µl of 10 µg ml^‐1^ FM4‐64 dye (Invitrogen, Waltham, MA, USA) solution dissolved in PBS. PBS was mixed with the dye in the same manner as the control for background signal. The sample‐dye mixtures were added to a black polystyrene 96 wells plate, and incubated for 15 min at room temperature while protected from light. The results were measured in a spectrophotometer at an excitation and emission wavelength of 515 and 640 nm respectively. After removing the background signals from all samples, a relative comparison was made between samples measured in the same assay.

### Transmission electron microscopy (TEM)

Negative staining was performed on EV samples prior to TEM imaging, where 2 μl of EV suspension was applied to a 400 mesh copper grid supplied with a formvar/carbon film. After 30 s of incubation, the grid was washed with 2 μl of milliQ water. Then, the grid was stained with 2% uranyl acetate for 30 s, and dried with filter paper. Samples were analysed with a Jeol JEM‐1400 plus TEM equipment (Jeol, Japan) with an accelerating voltage of 120 kV.

### Flow cytometry analysis

Prior to staining and flow cytometry analysis, the EV suspensions were treated with 1 mg ml^‐1^ DNase I (Roche) in a reaction buffer containing 10 mM tris–HCl (pH 7.5), 2.5 mM MgCl_2_ and 0.1 M CaCl_2_, at 30°C for 1 h. EVs were stained for flow cytometry with 10 µg ml^‐1^ FM4‐64 membrane dye and/or 16 µg ml^‐1^ DAPI DNA dye for 15 min at room temperature while protected from light. A BD‐FACS Aria III flow cytometer was used for flow cytometry analysis. Particles from the EV suspension were gated and differentiated based on forward scatter (FSC), side scatter (SSC), DAPI and FM4‐64 parameters. A 405 nm laser with a 502 LP filter (510/50 nm) was used to detect DAPI signals and a 561 nm laser with 780/60 nm filter for detection FM4‐64 signals. For each sample, 50 000 (non‐background) events were analysed.

BD FACSDiva™ software was used for data acquisition and FlowJo flow cytometry analysis software (version 10; Tree Star, Ashland, OR, USA) was applied for data analysis.

### Proteomics analysis

The EV samples were sonicated with a sonication probe two times for 30 s. Protein concentrations in EV and cytoplasmic membrane samples were determined by the bicinchoninic acid (BCA) assay. The protein aggregation capture (PAC) method (Batth *et al*., 2019) was used in a slightly modified way for preparation for proteomics analysis. In brief, in each sample, 60 µg of protein was reduced with 15 mM DTT at 45°C for 30 min, unfolded in 6 M Urea and alkylated with 20 mM acrylamide at room temperature for 30 min. The pH of the protein solution was adjusted to seven using 10% (v/v) trifluoro‐acetic acid (TFA). SpeedBeads (magnetic carboxylate modified particles, GE Healthcare) of product 45152105050250 and 65152105050250 were mixed with 1:1 ratio at 50 µg µl^‐1^, and 8 µl of SpeedBeads was added to each protein sample. Acetonitrile was added up to 71% (v/v) to the protein beads mixture, incubated at room temperature with gentle shaking for 20 min. A magnet was used to separate the SpeedBeads from the supernatant for 30 s, and the supernatant was removed. The SpeedBeads were then washed with 1 ml of 70% ethanol and 1 ml of 100% acetonitrile successively, resuspended in 100 µl of 5 ng µl^‐1^ trypsin solution and incubated overnight at room temperature with gentle shaking. The pH of SpeedBeads suspension was adjusted to three using 10% TFA, and the SpeedBeads were separated from the supernatant by using a magnet. The supernatant was filtered using C8 Empore disk filters. To improve yield, 0.1% formic acid was used to wash the beads and a 1:1 mixture of acetonitrile and 0.1% formic acid was used to wash the filter. All eluents were combined and dried to 10–15 µl, then topped up to 50 µl with 0.1% formic acid.

For the LC‐MS/MS analysis, 1.5 µl of prepared sample was injected into the system, and the analysis was performed essentially as described in Liu *et al*. (2021). The MaxQuant quantitative proteomics software package was used to analyse LC‐MS data with all MS/MS spectra as described previously (Cox *et al*., 2014), and the proteome of *L. lactis* ssp. *cremoris* TIFN1 (UniProt ID UP000015849) was used as the protein database. We used Perseus for filtering and further bioinformatics and statistical analysis of the MaxQuant ProteinGroups files (Tyanova *et al*., 2016). Reverse hits were removed; identified protein groups contained minimally two peptides, of which at least one is unique and one unmodified.

The values of intensity‐based absolute quantitation (iBAQ) was calculated by MaxQuant, in which the protein intensities are corrected for the number of measurable peptides.

### Bioinformatics analysis

For detecting DNA sequence similarities, the NCBI Blast‐n tool was used. For gene ontology (GO) annotation, the Uniprot Retrieve/ID mapping tool was used in combination with manual curation.

## Results

### The genome of strain *L. lactis* ssp. cremoris FM‐YL11

*Lactococcus lactis* ssp. *cremoris* FM‐YL11 was isolated from an artisanal cheese, and its genome was sequenced (GenBank No. CP071729). In the 2406538 bp chromosome of strain FM‐YL11, bases 1984716–2025964 were shown to share 100% sequence coverage and identity with prophage proPhi1 (GenBank No. MN534315) in *L. lactis* ssp. *cremoris* strain TIFN1 and proPhi5 (MN534318) from strain TIFN5 (Alexeeva *et al*., 2021), and 99.99% identity to corresponding chromosomal regions in *L. lactis* ssp. *cremoris* strain 3107 (GenBank No. CP031538) and JM4 (GenBank No. AP015909).

Sequencing of the prophage‐cured derivative FM‐YL12 (GenBank No. CP071728) confirmed that the prophage sequence in strain FM‐YL11 (bases 1984716–2025964) is absent in strain FM‐YL12, and this difference is the only gap when comparing sequences of the two strains. Except for the absence of the described prophage sequence, strain FM‐YL12 showed 99% sequences identity to strain FM‐YL11 with no rearrangement of the genome.

### *L. lactis* strain produces 10‐fold more EVs under a prophage‐inducing condition

**Fig. 1:**
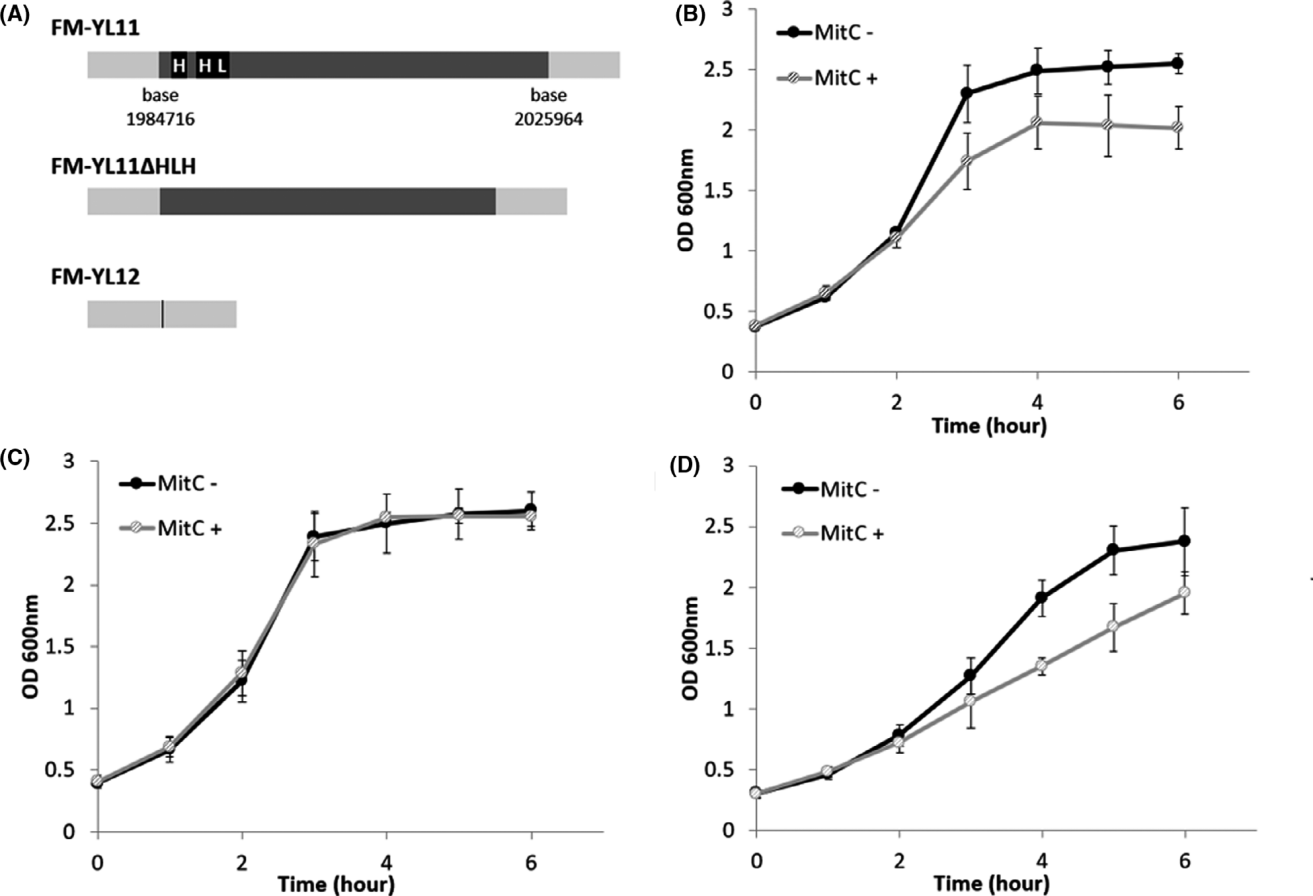
Strains of study and optical density‐based growth curves. A. Schematic presentation of the various strains used in this study. The bacterial chromosome (non‐prophage sequence) is shown in light grey, prophage shown in dark grey except for prophage holin and lysin genes shown in black. The insertion site of the prophage is shown as a black line for prophage‐cured strain FM‐YL12. Growth curves of (B) FM‐YL11; (C) FM‐YL12; (D) FM‐YL11ΔHLH under prophage‐inducing condition and non‐inducing condition based on OD_600_ values. For prophage‐inducing condition, 1 µg ml^‐1^ mitomycin C was added to bacterial cultures in early exponential phase. For non‐inducing condition, no mitomycin C was added to the cultures. Mitomycin C was added at time 0 h indicated by the graph. Data were from six independent experiments. Error bars show standard deviations.

To investigate the role of prophage in *L. lactis* EV production, as reported previously for *B. subtilis* (Toyofuku *et al*., 2017), two derivatives of strain FM‐YL11 were employed: strain FM‐YL12 is a prophage‐cured derivative of FM‐YL11, and FM‐YL11ΔHLH is a mutant in which the two holin genes and one endolysin gene encoded by the prophage have been knocked out (Fig. 1A). Prophage was induced by supplementing mitomycin C (MitC), an antibiotic causing DNA damage, which triggers prophage to enter the active lytic cycle in lysogenic bacteria (Oliveira *et al*., 2017; Filipiak *et al*., 2020), where prophage‐encoded genes are expressed, proteins synthesized and new phage particles formed. Typically, in the lytic cycle, the prophage‐encoded holin–lysin system degrades the cell envelope, leading to cell lysis and liberation of phage progenies.

Growth under non‐inducing and prophage‐inducing (by addition of 1 μg ml^‐1^ MitC) conditions of the wild‐type strain FM‐YL11 and the two derived mutants was compared (Fig. 1B and D). The prophage‐inducing condition did not result in massive cell lysis in the FM‐YL11 culture but resulted in a slight inhibition of growth (Fig. 1B). In addition, growth of strains FM‐YL12 (Fig. 1C) and FM‐YL11ΔHLH (Fig. 1D) was not and only slightly inhibited by the prophage‐inducing condition, respectively, as reflected by the growth curves of all strains with and without prophage induction. The growth behaviour of strain FM‐YL11 under both tested conditions was comparable to that reported for the lysogenic *L. lactis* ssp. *cremoris* strain TIFN1 (Alexeeva *et al*., 2018).

**Fig. 2:**
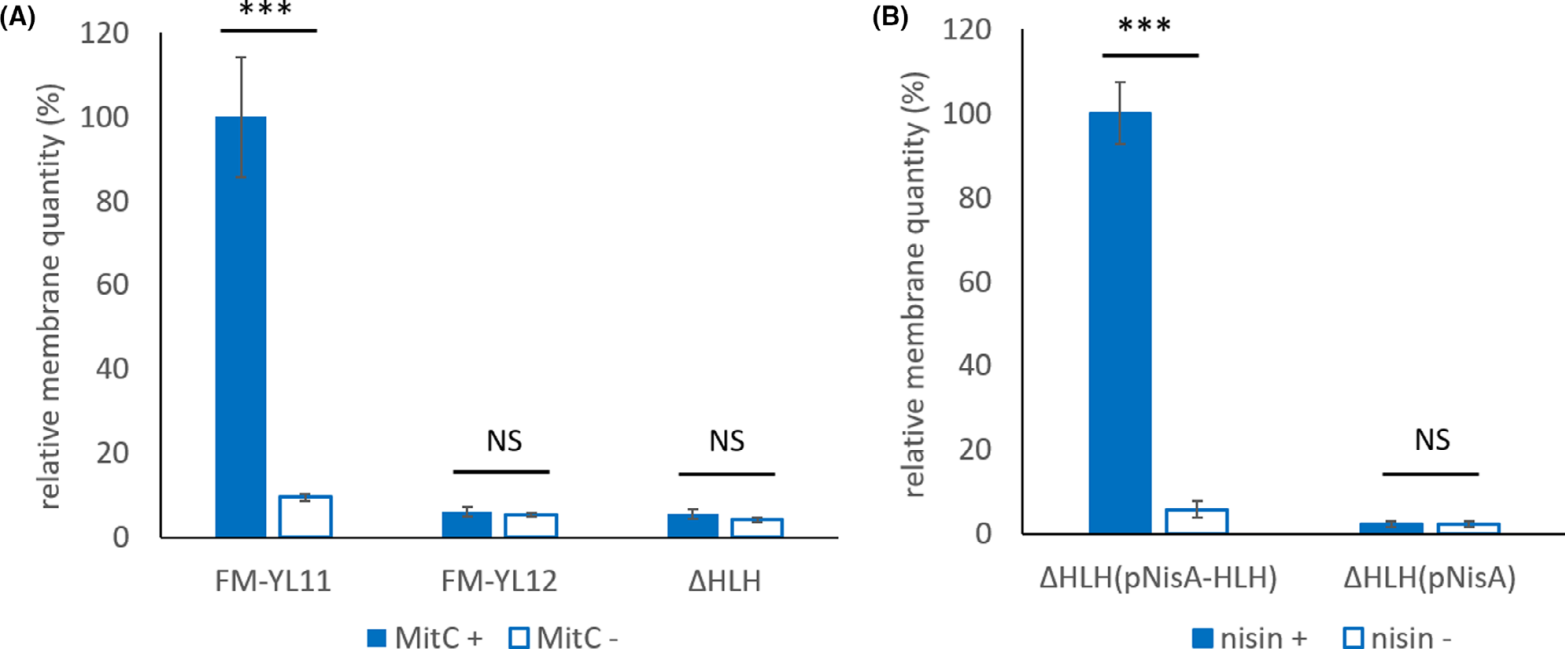
EV quantification by staining with membrane‐specific fluorescent dye FM4‐64. A. Relative membrane quantity of EVs isolated from the culture supernatant of strain FM‐YL11, FM‐YL12 and FM‐YL11ΔHLH (shown as ΔHLH) under prophage‐inducing condition and non‐inducing condition. The quantity (as reflected by the fluorescent signal intensity of dye FM4‐64) of EVs from FM‐YL11 under prophage‐inducing condition was regarded as 100%. For prophage‐inducing condition, 1 µg ml^‐1^ mitomycin C was added to bacterial cultures in early exponential phase, and EVs were collected from the supernatant by ultracentrifugation after 6 h of treatment. For non‐inducing condition, all treatments were the same except that no mitomycin C was added to the cultures. Samples were from four to six independent experiments. B. Relative membrane quantity of EVs isolated from the culture supernatant of FM‐YL11ΔHLH with holin–lysin gene complementation (pNisA‐HLH) and control plasmid (pNisA) under holin–lysin‐inducing condition and non‐inducing condition. The quantity (as reflected by the fluorescent signal intensity of dye FM4‐64) of EVs from FM‐YL11ΔHLH (pNisA‐HLH) under nisin‐inducing condition was regarded as 100%. For holin–lysin‐inducing condition, 10 ng ml^‐1^ nisin was added to bacterial cultures in early exponential phase, and EVs were collected from the supernatant by ultracentrifugation after 20 h of treatment. For non‐inducing condition, all treatments were the same except that no nisin was added to the cultures. Samples were from three to four independent experiments. Error bars show standard errors of the mean. ***, *P* < 0.001; NS, *P* > 0.05 in *t*‐tests.

Analysis of EV production under the tested conditions of strain FM‐YL11 and mutants at the early stationary phase showed the presence of structures with typical size and morphology of EVs in the recovered supernatant fractions (Fig. S1). In non‐inducing conditions, supernatant fraction of strain FM‐YL11 showed besides EV‐like structures, particles with typical size and morphology of phage heads (Fig. S1A and B). Under prophage‐inducing condition, the quantity of EVs recovered from FM‐YL11 culture supernatant was 10‐fold higher than in the culture without prophage induction, as reflected by fluorescent signals of membrane‐specific fluorescent dye (Fig. 2A). Again, EV‐like structures were observed in the fraction recovered from culture supernatant under the prophage‐induced condition, together with abundant phage head‐like particles (Fig. S1C and D). The sizes of EV‐like structures ranged from 50 nm to 300 nm in diameter, while the phage head‐like structures were around 50 nm in diameter.

### Prophage‐encoded holin–lysin system stimulates EV formation

When the strains FM‐YL12 and FM‐YL11ΔHLH were exposed to the prophage‐inducing condition, we did not observe an increase of the EV quantities recovered from culture supernatant, as compared with the non‐inducing condition (Fig. 2A). The EV quantities of both derivatives under both conditions were slightly lower than that from strain FM‐YL11 without prophage induction. EV‐like structures were observed in the EV fraction of both FM‐YL12 and FM‐YL11ΔHLH, and the phage head‐like structures could be spotted in the sample from YL11ΔHLH but no longer in FM‐YL12 (Fig. S1E–H). The EV‐like structures recovered from FM‐YL12 were in general smaller than 150 nm in diameter.

To verify the role of prophage‐encoded holin–lysin system in EV production of *L. lactis*, we constructed a mutant with holin and lysin gene complementation. Mutant FM‐YL11ΔHLH was transformed with a plasmid containing the prophage encoded two holins and one endolysin gene, using a nisin‐inducible promoter (pNisA‐HLH) to drive expression. Addition of nisin resulted in 10‐fold increase of EV production in the mutant with holin–lysin gene complementation but not in the control strain where only an empty vector (pNisA) was introduced (Fig. 2B).

### A subpopulation of holin–lysin‐induced EVs enclose tailless phage particles

We examined the composition and cargo of the isolated EV fractions from strain FM‐YL11 under the prophage‐inducing condition. The EV fractions were first treated with DNase to remove any extra‐vesicular DNA, and then stained with fluorescent dyes for flow cytometry analysis. EVs from FM‐YL12 treated under the same condition served as the phage‐free control.

**Fig. 3:**
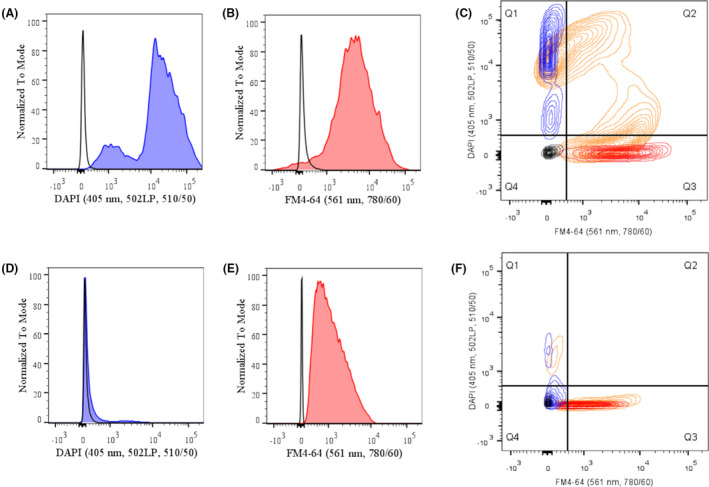
Flow cytometry analysis of EVs stained by DNA and membrane‐specific fluorescent dyes. (A–C) are analysis of EVs from FM‐YL11 under prophage‐inducing condition, while (D–F) are analysis of EVs from FM‐YL12 under prophage‐inducing condition. A. and D. fluorescence distribution in the population of EVs stained by DNA dye DAPI (blue filled) comparing to unstained EVs (black unfilled). B. and E. fluorescence distribution in the population of EVs stained by membrane‐specific dye FM4‐64 (red filled) comparing to unstained EVs (black unfilled). C. and F. superimposed signal distribution of EVs that are unstained (black), stained only with DAPI (blue), stained only with FM4‐64 (red), and stained with DAPI and FM4‐64 simultaneously (orange).

The EVs from strain FM‐YL11 stained with the DNA dye DAPI showed clear positive signals for all analysed particles compared with the unstained samples (Fig. 3A), while the majority of FM‐YL12 EVs showed no signal upon DNA staining except for a very small population showing a low DNA signal (Fig. 3D). We identified two distinct populations varying in signal intensity in the EV fraction of strain FM‐YL11 (Fig. 3A). The population with relatively low DNA signal intensity, but not the population with very high signal intensity, was observed in the EV fraction from the prophage‐cured derivative FM‐YL12 (Fig. 3D). Based on EM pictures, phage head‐like particles were spotted in the EV fraction from FM‐YL11 (Fig. S1C and D). It is plausible that the very high DNA signal was achieved by the tight packing of the phage genome. Therefore, we deduced that the population with very high DNA signal intensity consisted of the phage heads.

When stained with the membrane‐specific dye FM4‐64, EVs from FM‐YL12 all showed positive signals compared with the unstained sample (Fig. 3E). A small population of FM‐YL11 EVs showed negative signals for the membrane staining, while the majority of the EVs were positive (Fig. 3B). The average signal intensity in FM‐YL12 sample was lower than the membrane‐positive population of FM‐YL11. This could be caused by the difference in EV sizes in the two samples, as also observed by electron microscopy (Fig. S1).

When the EV fraction from strain FM‐YL11 was stained with the DNA and membrane dye simultaneously, the majority of the population with low DNA signals showed positive signals for the membrane dye (Fig. 3C, lower orange cloud in Q2), indicating a population of EVs enclosing free DNA molecules. This was hardly observed from FM‐YL12, where the majority EVs showed positive membrane signal but low/negative for DNA signal with the double staining (Fig. 3F).

**Fig. 4:**
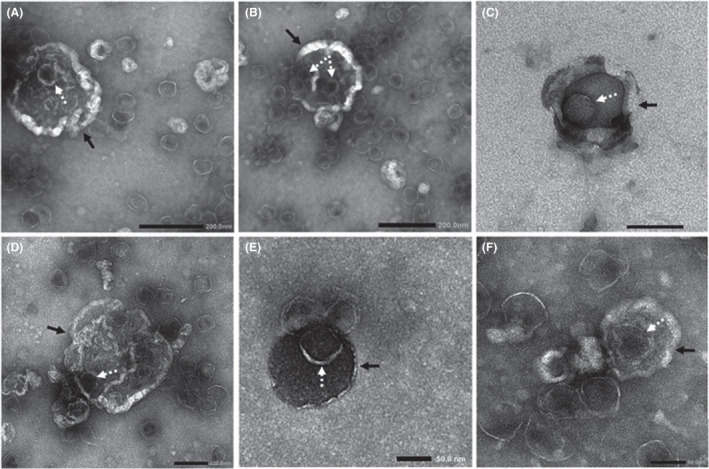
TEM pictures of EVs isolated from FM‐YL11 supernatant under prophage‐inducing condition. Cases are shown where phage particles seemed to be enclosed in EVs. Arrows with solid lines point at EV‐like structures, and arrows with dashed lines point at phage head‐like particles. Scale bars in (A) and (B) represent 200 nm, in (C) and (D) represent 100 nm and in (E) and (F) 50 nm.

The population with very high DNA signals in FM‐YL11 was divided further into a subpopulation that is high in membrane signal (Fig. 3C, higher orange cloud in Q2) and a subpopulation low/negative in membrane signal (Fig. 3C, orange cloud in Q1). The subpopulation high in DNA signal but low/negative in membrane signal likely consisted of the free, unenveloped phage heads, while the subpopulation high in both DNA and membrane signal likely pointed to EVs that enclosed tailless phage particles. Structures that conceivably represent the subpopulation of EVs enclosing phage heads were also observed under electron microscope (Fig. 4).

### Proteome of holin–lysin‐included EVs

The proteome of the EV fraction collected from the culture supernatant of strain FM‐YL11 under prophage‐inducing condition was examined. The reference genome used for FM‐YL11 contained 2670 predicted proteins, and 1283 proteins were detected in the EV samples. Average iBAQ (intensity‐based absolute quantification) values of samples from two independent experiments were examined. Due to very high sensitivity of the detection method as well as limitations in the EV purification method, here we only focus on the top 600 proteins in abundance (Table S3) to describe the main proteome profile, which counted up to 98% of the total protein quantity.

Phage head protein and scaffolding protein were among the most abundant proteins revealed from the FM‐YL11 EV fraction, counting up to 11% signal intensity of the total top 600 proteins, further confirming the identity of the phage head‐like structures observed next to EVs. Phage tail proteins were also detected in the top 600 protein list in the EV fraction from FM‐YL11, although we did not observe any phage tail‐like structures in TEM analysis. In total, about 40 proteins encoded by the prophage in FM‐YL11, including holin and lysin were also among the top 600 most abundant proteins found in the EV fraction of FM‐YL11.

**Fig. 5:**
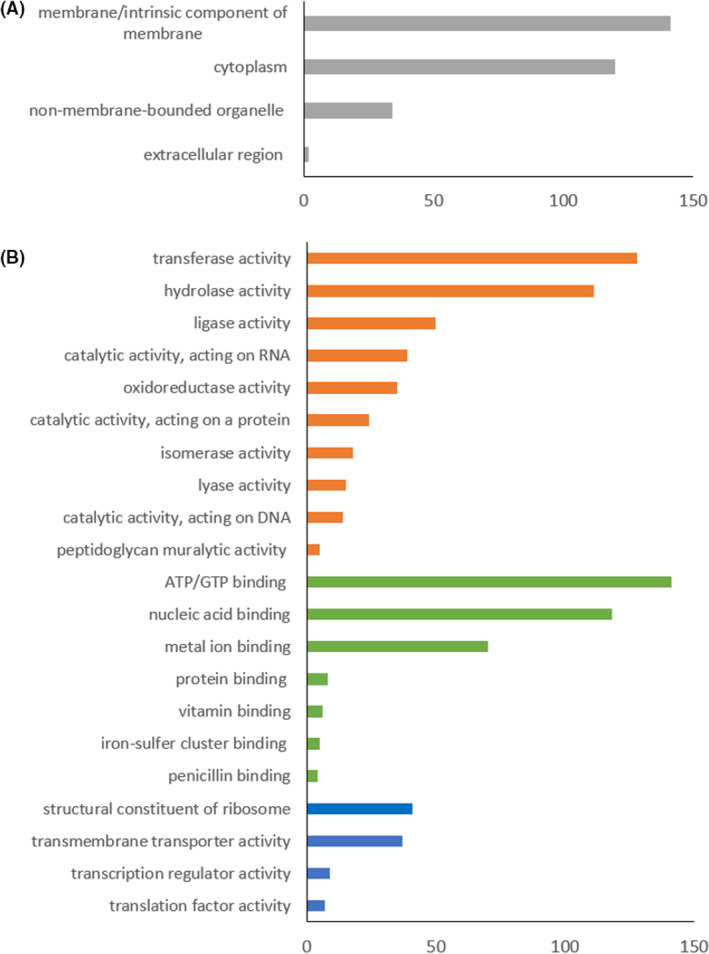
Distribution of top 500 proteins from *L. lactis* FM‐YL11 prophage holin–lysin induced EVs based on their GO annotations in (A) subcellular locations and (B) molecular functions. In (B) orange bars represent subcategories under category ‘catalytic activity’, green bars represent subcategories under ‘binding’ and blue bars for other categories under the domain of molecular functions. Some proteins are assigned to more than one category.

When examining the gene ontology (GO) annotations of the top 600 proteins detected in the EV fraction of FM‐YL11, more than 300 proteins were assigned a GO term as cellular anatomical entity under the cellular component domain, from which more than 140 proteins were assigned a GO term of membrane, and 120 proteins were assigned to be in the cytoplasm (Fig. 5A). Nearly 40 ribosomal proteins also made a significant category. For molecular function, more than 320 proteins were assigned a GO term of catalytic activity, 280 proteins with binding activity and about 40 proteins with transmembrane transporter activity. Note that it is possible for one protein to get multiple GO terms (Fig. 5B). Among the proteins with catalytic activity, transferase and hydrolase activities were the most abundant with more than 100 hits. Among the proteins with binding activity, ATP/GTP binding, nucleic acid binding and metal ion binding (magnesium, zinc, iron and potassium binding) were the subcategories with the most hits. The proteins in FM‐YL11 EVs are predicted to be involved in a variety of biological processes according to the GO annotation, including translation, localization, cell division, lipid biosynthesis, peptidoglycan biosynthesis, stress/stimulus response, etc (Table S3).

## Discussion

Recent insight in variety in cargos carried by Gram‐positive EVs and the diverse roles they play in bacterial physiology, ecology and microbe–host interactions highlighted the importance of this research field. Gram‐positive bacterial species cover a wide range of ecological roles including those of beneficial commensal bacteria and pathogens. Hence, knowledge on the biogenesis, composition and functions of Gram‐positive EVs contributes not only to fundamental research in microbiology, but also to understanding the implications of Gram‐positive EV in human health and disease, and to diverse potential biotechnological or medical applications (Liu *et al*., 2018; Briaud and Carroll, 2020).

In this study, we provide further evidence for EV production by the Gram‐positive bacterium *Lactococcus lactis*, using an artisanal cheese isolate FM‐YL11 as a model. We showed that *L. lactis* FM‐YL11 EV production was largely stimulated by prophage activity, and more specifically, by the prophage‐encoded holin–lysin system. Defective phage particles were found to be released along with and enclosed by EVs.

Prophage‐dependent EV release mechanism has also been demonstrated in other Gram‐positive bacteria, namely, *Bacillus subtilis* and *Staphylococcus aureus* (Toyofuku *et al*., 2017; Andreoni *et al*., 2019). The prophage‐triggered EV release in *B. subtilis* was not found to cause immediate cell death and explosive cell lysis: the cells remained in shape during EV extrusion, although eventually cell death took place as the integrity of the cytoplasmic membrane was heavily compromised. Phage particles (with tails) were found to be enclosed by the *B. subtilis* EVs. In *S. aureus* the prophage‐dependent mechanism of EV release was found to be associated with cell lysis upon prophage activation, as indicated by ghost cells and (tailed) phage particles released along with the EVs. Observed differences in lysis of *B. subtilis* and *S. aureus* producer cells may be explained by extend of prophage induction and differences in activity of prophage‐encoded cell envelop degrading enzymes.

**Fig. 6:**
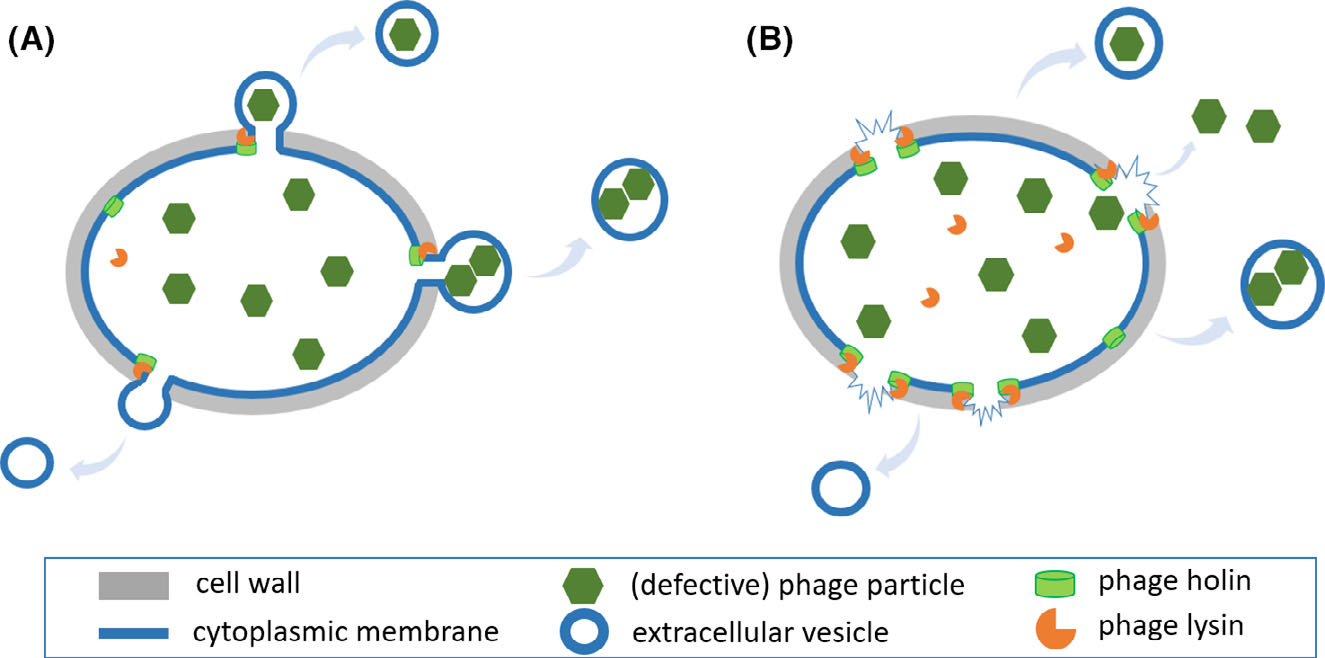
Schematic presentation of the proposed model for prophage holin–lysin induced EV production mechanism in *L. lactis*. A. When prophage‐encoded holin and lysin are produced at moderate levels in *L. lactis* under the tested prophage‐inducing condition, EVs are produced by gradual extrusion of the cytoplasmic membrane without immediate cell lysis. This is expected to be the case for the major bacterial population in the culture of strain FM‐YL11. The (defective) phage particles accumulated in the cytoplasm may be enclosed by EVs upon release. B. When prophage‐encoded holin and lysin are produced at high levels in *L. lactis* under the tested prophage‐inducing condition, EVs are produced by cell lysis. This scenario is expected to be among a small subpopulation in the culture of strain FM‐YL11. Phage particles are released freely into the extracellular environment or enclosed by the membrane upon forming EVs. Note that not all illustrated components are drawn at the same scale.

Combining all information, we propose a new model for EV production in *L. lactis* that is a mixed scenario of the gradual extrusion/non‐lytic release and cell lysis as a result of prophage holin–lysin activity (Fig. 6). Under prophage‐inducing condition, the growth of FM‐YL11 appeared to be only slightly inhibited in comparison with the non‐inducing condition. The same effect of slight growth inhibition was observed for FM‐YL11ΔHLH under the prophage‐inducing condition but not for FM‐YL12, indicating that this growth inhibition was most likely a result of the metabolic burden for producing intracellularly accumulating phage particles instead of cell lysis during EV production. Other than the growth inhibition, it is likely that the majority of FM‐YL11 cells remained intact throughout the 6 h following exposure to mitomycin C, in spite of substantial release of EVs. The presence of phage particles in the culture supernatant may point to cell lysis of a small subpopulation. In the presented model, the gradual extrusion of EVs and cell lysis formed EVs were due to the action of prophage‐encoded holin and lysin. Differences in EV production routes may be explained by heterogeneity in prophage gene expression and production of phage proteins in the population upon prophage induction, with the subpopulation of lysed cells representing cells with high level holin–lysin production (Fig. 6B), while the majority of cells are considered to have a moderate level of production that allows for gradual, non‐lytic release of EVs (Fig. 6A).

Although EV production was clearly stimulated by prophage‐encoded holin–lysin system in *L. lactis*, prophage‐independent EV production can also occur. This is reflected by the presence of EVs in the culture supernatants from both the non‐inducing and prophage‐inducing conditions of strains FM‐YL12 and YL11 ΔHLH, albeit in low quantity. Prophage‐independent triggers have also been described for EV production in *S. aureus*, for example, antibiotics that weaken the peptidoglycan layer and production of cell wall degrading autolysins (Wang *et al*., 2018; Andreoni *et al*., 2019). *L*. *lactis* is known to produce autolysins (N‐acetylglucosaminidases) too. The four lactococcal autolysins, namely, AcmA, AcmB, AcmC and AcmD, have been experimentally confirmed for their functionality in cell separation and autolysis in the model strain *L. lactis* ssp. *cremoris* MG1363 (Buist *et al*., 1997; Visweswaran *et al*., 2013, 2017). In the genome of strain FM‐YL11, we identified homologous sequences of all four autolysin‐encoding genes to strain MG1363 (GenBank No. AM406671) with 97–98% sequences identity. A possible role of putative autolysins in EV production in *L. lactis* remains to be confirmed.

In comparison with the EVs produced by strain FM‐YL12, the prophage‐encoded holin–lysin induced EVs in strain FM‐YL11 seem to be larger in size, as reflected by both electron microscopy and flow cytometry of membrane‐stained particles. Besides the subpopulation of EVs from strain FM‐YL11 that enclosed phage particles with uniquely high DNA content, most likely there is also an EV subpopulation that entrapped a substantial amount of other DNA molecules. Prophage holin–lysin‐induced EVs generally show a high DNA content in comparison with EVs produced by a prophage‐independent route. Similar observations were reported in the study of *S. aureus* EV production via phage‐dependent and ‐independent routes, conceivably as a result of fragmentation of the bacterial chromosome caused by prophage activation (Andreoni *et al*., 2019). Proteomics analysis in our study also revealed a number of nucleic acid binding proteins in EVs released by strain FM‐YL11. Our finding of nucleic acid cargos in *L. lactis* EVs adds to the limited evidence that Gram‐positive EVs contain nucleotides as the cargo (Klieve *et al*., 2005; Resch *et al*., 2016; Rodriguez and Kuehn, 2020). DNA containing EVs potentially facilitate the exchange of genetic material between bacterial cells in a microbial community.

Moreover, the substantial population of holin–lysin‐induced EVs that contain phage head‐like particles is intriguing. This deduction was supported by EM pictures and flow cytometry. In the latter, a population with positive membrane signals and distinctly high DNA signals was identified. Notably, in the study of the lysogenic strain TIFN1, when phage particles were collected from similar prophage induction conditions, the complete proPhi genome was identified with high DNA sequencing coverage in the phage particles (Alexeeva *et al*., 2021). In addition, the prophage sequence in strain FM‐YL11 showed 100% sequence identity to the *Siphoviridae* phage proPhi1 (Alexeeva *et al*., 2021), where the tail tape measurement gene was found to be truncated by a mobile element, explaining the tailless phenotype of the phages. Interestingly, viral particles or complete viral genomes have been previously observed in *B. subtilis* and *Thermococcus nautilus* EVs and roles in exchange of genetic material have been suggested (Gaudin *et al*., 2014; Kim *et al*., 2016; Toyofuku *et al*., 2017). In addition, Tzipilevich *et al*. (2017) have demonstrated that *B. subtilis* EVs may contribute to phage infection by spreading phage receptors to non‐sensitive strains and making them susceptible to phage infection. Similarly, proteomics analysis also revealed phage infection protein (T0UL74) in FM‐YL11 EVs (Table S3), which may potentially enable EVs to act as decoy for phage targeting or spread phage sensitivity to other cells. Whether the EVs enclosing phage particles offer a novel route in lactococcal phage infection remains to be studied.

Proteomic analysis of holin–lysin‐induced EVs showed high abundance of membrane proteins and cytoplasmic proteins, the latter indicating enclosure of cytoplasmic content by EVs, in line with previous studies of other Gram‐positive EVs (Lee *et al*., 2009; Kim *et al*., 2016). This is considered a unique feature for Gram‐positive EVs, in contrast to Gram‐negative EVs, since EVs from Gram‐positive bacteria are generated from the cytoplasmic membrane allowing direct encapsulation of cytoplasmic content, while Gram‐negative EVs are mostly generated from the outer membrane, and tend to encapsulate periplasmic components (Kim *et al*., 2015). It remains to be elucidated whether the proteins in EVs were loaded randomly or by specific sorting mechanisms generated by specific cellular domains in the cytoplasmic membrane of *L. lactis* strain FM‐YL11. Protein, lipid and nucleic acid analysis of bacterial EVs from *Streptococcus pyogenes*, *Streptococcus mutans*, *S. aureus* and *Lactiplantibacillus plantarum* also revealed compositional differences compared with the respective cell membranes (Lee *et al*., 2009; Liao *et al*., 2014; Biagini *et al*., 2015; Kim *et al*., 2020a), suggesting that active EV production involves dedicated sorting mechanisms. In this study, the over‐representation of proteins involved in translation, lipid and peptidoglycan biosynthesis, and cell division in FM‐YL11 EVs (Table S3), may point to hotspots for EV production, such as the cell division site. Similarly, in the previous study of membrane‐enclosed phage release by *L. lactis* isolates from the starter culture Ur, EV release was most prominent at the cell division site, and had a lipid profile that differed from that of the cytoplasmic membrane (Liu *et al*., 2022).

Protein cargos detected in the holin–lysin‐induced *L. lactis* EVs also included metal ion (e.g. iron) binding proteins and penicillin‐binding proteins (Table S3). Similarly, EVs from *Mycobacterium tuberculosis*, *Streptomyces coelicolor* and *S*. *aureus* were shown to contain iron‐binding factors, which contributed to bacterial survival under iron‐limited conditions (Lee *et al*., 2009, 2015; Schrempf *et al*., 2011). The penicillin‐binding proteins are involved in peptidoglycan synthesis and cell division (David *et al*., 2018), but also offer binding sites to penicillin and β‐lactam antibiotics. This observation supports previous findings where EVs have been considered to contribute to bacterial survival by removing the antibiotics from the extracellular environment, acting as the decoy of antibiotic targeting or containing antibiotic‐degrading enzymes (Lee *et al*., 2009; Chattopadhyay and Jaganandham, 2015; Kim *et al*., 2018; Sabnis *et al*., 2018; Bose *et al*., 2020; Kim *et al*., 2020b).

Besides the solid evidence on the roles of prophage holin–lysin in *L. lactis* EV production, this study also highlights the interest for further investigations to answer the remaining important questions: first, comparative analysis of the nucleic acid and protein content in EVs and producer cells will support validation of the hypothesized model of EV release (Fig. 6), and the occurrence of subpopulations of EVs, as well as to providing insights on the possible physiological and ecological significance of EV production. Second, mechanisms underlying prophage induction and hence EV production can be further investigated in relation to possible DNA damage and RecA‐mediated damage repair as described for *B. subtilis* (Toyofuku *et al*., 2017). Last but not least, it is of high interest to examine the effect of prophage activation and EV release on colonization capacity and competitive fitness of respective producer(s) in selected environments.

To conclude, this study adds *L*. *lactis* to the list of EV‐producing Gram‐positive bacteria species. Evidence is provided for an important role of phage holin–lysin in EV release. As prophages or prophage‐encoded elements are extremely widespread and common in bacterial genomes, the role of prophage activation, especially the holin–lysin system, in EV production can be generalized to more Gram‐positive bacteria. Considering the important role of *L. lactis* and other LAB in a range of fermentations and probiotic formulations, knowledge of the EV production mechanisms can be exploited to achieve desired traits and functionalities in fermentation processes, such as efficient release and delivery of intracellular/membrane embedded effector molecules or nutritional compounds, and in probiotic applications. Moreover, findings from this study can also pave the way for exploiting bacterial EVs for biotechnological applications, such as for delivery of genome editing tools (Liu *et al*., 2019).

## Conflict of interest

The authors declare that the research was conducted in the absence of any commercial or financial relationships that could be construed as a potential conflict of interest.

## Supporting information


**Fig. S1**. TEM pictures of lactococcal EVs. A) and B) are EVs collected from the culture supernatant of strain FM‐YL11, under non‐inducing condition. C) and D) are EVs from the culture supernatant of strain FM‐YL11, under prophage‐inducing condition. E) and F) are EVs from strain FM‐YL12 supernatant under prophage‐inducing condition. G) and H) are from strain FM‐YL11ΔHLH, under prophage‐inducing condition. For prophage‐inducing condition, 1 µg ml^‐1^ mitomycin C was added to bacterial cultures in early exponential phase, and EVs were collected from the supernatant by ultracentrifugation after 6 h treatment. For non‐inducing condition, all treatments were the same except that no mitomycin C was added to the cultures. Arrows with solid lines point at EV‐like structures, and arrows with dashed lines point at phage head‐like particles. All scale bars represent 200 nm.
**Table S1**. Primers used for constructing plasmids used for holin and lysin gene knockout in strain FM‐YL11.
**Table S2**. Primers used for constructing plasmids used for holin and lysin gene complementation in strain FM‐YL11ΔHLH.
**Table S3**. Top 600 protein hits with greatest abundance from the EV fraction of strain FM‐YL11 under the prophage‐inducing condition. Samples were from two independent experiments. Seven GO categories were selected to be presented here: 1 ‐ membrane (associated) protein, 2 – prophage encoded protein, 3 – translation, 4 – cell division, 5 – lipid biosynthesis, 6 – peptidoglycan biosynthesis, 7 – stress/stimulus response.
