# Supplementary material for: Extracellular vesicle formation in Lactococcus lactis is stimulated by prophage‐encoded holin–lysin system
Source: Microb Biotechnol. 2022 Mar 1;15(4):1281–95. doi: 10.1111/1751-7915.13972 (PMC8966010; doi:10.1111/1751-7915.13972)
Supplement: Supplementary file 1 — Fig. S1. TEM pictures of lactococcal EVs. A) and B) are EVs collected from the culture supernatant of strain FM‐YL11, under non‐inducing condition. C) and D) are EVs from the culture supernatant of strain FM‐YL11, under prophage‐inducing condition. E) and F) are EVs from strain FM‐YL12 supernatant under prophage‐inducing condition. G) and H) are from strain FM‐YL11ΔHLH, under prophage‐inducing condition. For prophage‐inducing condition, 1 µg ml‐1 mitomycin C was added to bacterial cultures in early exponential phase, and EVs were collected from the supernatant by ultracentrifugation after 6 h treatment. For non‐inducing condition, all treatments were the same except that no mitomycin C was added to the cultures. Arrows with solid lines point at EV‐like structures, and arrows with dashed lines point at phage head‐like particles. All scale bars represent 200 nm. Table S1. Primers used for constructing plasmids used for holin and lysin gene knockout in strain FM‐YL11. Table S2. Primers used for constructing plasmids used for holin and lysin gene complementation in strain FM‐YL11ΔHLH. Table S3. Top 600 protein hits with greatest abundance from the EV fraction of strain FM‐YL11 under the prophage‐inducing condition. Samples were from two independent experiments. Seven GO categories were selected to be presented here: 1 ‐ membrane (associated) protein, 2 – prophage encoded protein, 3 – translation, 4 – cell division, 5 – lipid biosynthesis, 6 – peptidoglycan biosynthesis, 7 – stress/stimulus response. [file MBT2-15-1281-s001.docx]

**Supplementary materials**


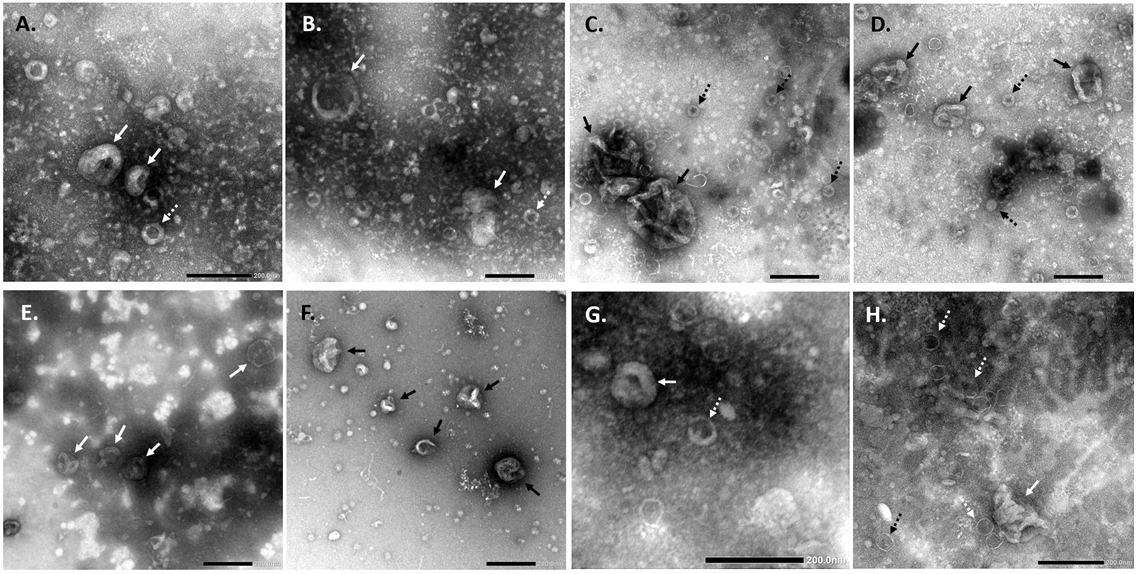


**Figure S1. TEM pictures of lactococcal EVs.** A) and B) are EVs collected from the culture supernatant of strain FM-YL11, under non-inducing condition. C) and D) are EVs from the culture supernatant of strain FM-YL11, under prophage-inducing condition. E) and F) are EVs from strain FM-YL12 supernatant under prophage-inducing condition. G) and H) are from strain FM-YL11ΔHLH, under prophage-inducing condition. For prophage-inducing condition, 1 µg/mL mitomycin C was added to bacterial cultures in early exponential phase, and EVs were collected from the supernatant by ultracentrifugation after 6 hours treatment. For non-inducing condition, all treatments were the same except that no mitomycin C was added to the cultures. Arrows with solid lines point at EV-like structures, and arrows with dashed lines point at phage head-like particles. All scale bars represent 200 nm.

**Table S1 Primers used for constructing plasmids used for holin and lysin gene knockout in strain FM-YL11.**

| Name | Sequence (5’-3’) |
| --- | --- |
| HL_up_XhoI Fw | GGTGGTCTCGAG**ACGGTTTCACGACTGTTTATCCG** |
| HL_up_HindIII Rv | CCTCCTAAGCTT**ATGTTTTTATGCAGTCCGTTGCC** |
| HL_dn_HindIII Fw | CTCCTCAAGCTT**AGAAATACCCTTGGCATATTCGC** |
| HL_dn_PstI Rv | CCACCACTGCAG**CCAAAAGCTCCATATTGTTCACC** |
| Holin_up PstI Rv | CCGCCGCTGCAG**GATCCCTACTTTCTTAATCTATTG** |
| Holin_up_XhoI-Fw | ATATATCTCGAG**CTCAAGCAAGTACGATTGGG** |
| holin1-dn HR_PstI Fw | GAGGAGCTGCAG**AAGGAGTTCCCAATGAGTTTAG** |
| holin1_dn HR_EagI Rv | CCTCCTCGGCCG**GTCTGTCATTGCACTTTGTAAGC** |

**Table S2 Primers used for constructing plasmids used for holin and lysin gene complementation in strain FM-YL11ΔHLH.**

| Name | Sequence (5’-3’) |
| --- | --- |
| 45-holin-lysin_fwd | CACTCACCATGGGTACTGCAG**TAGGAGAGTAAAATGAATCAAATC** |
| 45/35-holin-lysin_rev | TCCCTACTTT**GCCAAGGGTATTTCTTTAATAAAC** |
| 45/35-holin2_fwd | TACCCTTGGC**AAAGTAGGGATCATGGAGG** |
| 45-holin2_rev | TCAAAGAAAGCTTGAGCTCT**GGGAACTCCTTTTAAAGTATTTTAG** |

**Table S3. Top 600 protein hits with greatest abundance from the EV fraction of strain FM-YL11 under the prophage-inducing condition.** Samples were from two independent experiments. Seven GO categories were selected to be presented here: 1 - membrane (associated) protein, 2 – prophage encoded protein, 3 – translation, 4 – cell division, 5 – lipid biosynthesis, 6 – peptidoglycan biosynthesis, 7 – stress/stimulus response.

| **UniProt IDs** | **Protein names** | **iBAQ signal** | **GO categories** | | | | | | |
| --- | --- | --- | --- | --- | --- | --- | --- | --- | --- |
|  |  |  | **1** | **2** | **3** | **4** | **5** | **6** | **7** |
| T0UW71 | Head protein | 6.29E+09 |  |  |  |  |  |  |  |
| T0URE1 | Elongation factor Tu | 3.45E+09 |  |  |  |  |  |  |  |
| T0UW80 | Scaffolding protein | 2.59E+09 |  |  |  |  |  |  |  |
| T0W8S4 | 50S ribosomal protein L4 | 2.55E+09 |  |  |  |  |  |  |  |
| T0W8S8 | 30S ribosomal protein S3 | 1.65E+09 |  |  |  |  |  |  |  |
| T0UZ71 | 30S ribosomal protein S11 | 1.57E+09 |  |  |  |  |  |  |  |
| T0UIU4 | 30S ribosomal protein S4 | 1.4E+09 |  |  |  |  |  |  |  |
| T0VMR3 | 30S ribosomal protein S7 | 1.3E+09 |  |  |  |  |  |  |  |
| T0V5G7 | 30S ribosomal protein S2 | 1.22E+09 |  |  |  |  |  |  |  |
| T0W643 | 50S ribosomal protein L5 | 1.21E+09 |  |  |  |  |  |  |  |
| T0UYK9 | 50S ribosomal protein L3 | 1.2E+09 |  |  |  |  |  |  |  |
| T0UT97 | 50S ribosomal protein L2 | 1.16E+09 |  |  |  |  |  |  |  |
| T0VXP0 | 50S ribosomal protein L19 | 1.13E+09 |  |  |  |  |  |  |  |
| T0VSC0 | 30S ribosomal protein S12 | 1.1E+09 |  |  |  |  |  |  |  |
| T0W645 | 30S ribosomal protein S5 | 1.08E+09 |  |  |  |  |  |  |  |
| T0UZG8 | Universal stress protein UspA | 1.01E+09 |  |  |  |  |  |  |  |
| T0VRD6 | Peptide-binding protein | 1.01E+09 |  |  |  |  |  |  |  |
| T0UZC3 | Preprotein translocase subunit YajC | 1E+09 |  |  |  |  |  |  |  |
| T0URV5 | Foldase protein PrsA | 1E+09 |  |  |  |  |  |  |  |
| T0UTB9 | 30S ribosomal protein S13 | 9.17E+08 |  |  |  |  |  |  |  |
| T0W5P3 | UPF0154 protein LLT1_9680 | 9.05E+08 |  |  |  |  |  |  |  |
| T0UQ99 | 50S ribosomal protein L21 | 8.86E+08 |  |  |  |  |  |  |  |
| T0URM1 | 50S ribosomal protein L20 | 7.28E+08 |  |  |  |  |  |  |  |
| T0UM40 | 30S ribosomal protein S1 | 6.62E+08 |  |  |  |  |  |  |  |
| T0VRG6 | Galactose-6-phosphate isomerase subunit LacB | 6.22E+08 |  |  |  |  |  |  |  |
| T0VR76 | Membrane protein | 6.16E+08 |  |  |  |  |  |  |  |
| T0W1W8 | ATP synthase subunit beta | 5.97E+08 |  |  |  |  |  |  |  |
| T0UPF6 | CTP synthase | 5.87E+08 |  |  |  |  |  |  |  |
| T0URD6 | Uncharacterized protein | 5.7E+08 |  |  |  |  |  |  |  |
| T0VVL7 | Dihydrolipoamide acetyltransferase component of pyruvate dehydrogenase complex | 5.62E+08 |  |  |  |  |  |  |  |
| T0W2N8 | Portal protein | 5.32E+08 |  |  |  |  |  |  |  |
| T0W640 | 50S ribosomal protein L16 | 5.32E+08 |  |  |  |  |  |  |  |
| T0W4T5 | UTP--glucose-1-phosphate uridylyltransferase | 5.28E+08 |  |  |  |  |  |  |  |
| T0W8U7 | 50S ribosomal protein L17 | 5.14E+08 |  |  |  |  |  |  |  |
| T0UKQ3 | Uncharacterized protein | 5.14E+08 |  |  |  |  |  |  |  |
| T0URG4 | 50S ribosomal protein L35 | 5.12E+08 |  |  |  |  |  |  |  |
| T0W5S1 | [neck passage]BppU_N domain-containing protein | 4.69E+08 |  |  |  |  |  |  |  |
| T0UZ62 | 30S ribosomal protein S8 | 4.47E+08 |  |  |  |  |  |  |  |
| T0W275 | Methionine ABC transporter ATP-binding protein | 4.43E+08 |  |  |  |  |  |  |  |
| T0URE6 | 30S ribosomal protein S15 | 4.23E+08 |  |  |  |  |  |  |  |
| T0UYM1 | 50S ribosomal protein L6 | 3.97E+08 |  |  |  |  |  |  |  |
| T0UA69 | EIICB-Lac | 3.97E+08 |  |  |  |  |  |  |  |
| T0VS84 | 30S ribosomal protein S9 | 3.87E+08 |  |  |  |  |  |  |  |
| T0UZ53 | 30S ribosomal protein S10 | 3.64E+08 |  |  |  |  |  |  |  |
| T0UKP1 | ATP synthase subunit alpha | 3.61E+08 |  |  |  |  |  |  |  |
| T0VTL1 | Enoyl-[acyl-carrier-protein] reductase [NADH] | 3.58E+08 |  |  |  |  |  |  |  |
| T0W2V1 | PTS fructose transporter subunit IIC | 3.55E+08 |  |  |  |  |  |  |  |
| T0UYS4 | GTP-binding protein | 3.51E+08 |  |  |  |  |  |  |  |
| T0USD3 | PTS mannose transporter subunit IID | 3.44E+08 |  |  |  |  |  |  |  |
| T0W2P1 | [tail length tape-measure protein] Uncharacterized protein | 3.37E+08 |  |  |  |  |  |  |  |
| T0UZ58 | 30S ribosomal protein S17 | 3.37E+08 |  |  |  |  |  |  |  |
| T0UVN1 | Uncharacterized protein | 3.3E+08 |  |  |  |  |  |  |  |
| T0UI30 | Protease | 3.24E+08 |  |  |  |  |  |  |  |
| T0UY81 | 50S ribosomal protein L1 | 3.13E+08 |  |  |  |  |  |  |  |
| T0VRU5 | Peptide ABC transporter ATP-binding protein | 3.08E+08 |  |  |  |  |  |  |  |
| T0UQG3 | ATP-dependent zinc metalloprotease FtsH | 3.02E+08 |  |  |  |  |  |  |  |
| T0URA1 | Uncharacterized protein | 2.98E+08 |  |  |  |  |  |  |  |
| T0UWL2 | S-adenosylmethionine synthase | 2.97E+08 |  |  |  |  |  |  |  |
| T0USJ1 | Catabolite control protein A | 2.96E+08 |  |  |  |  |  |  |  |
| T0W634 | Uncharacterized protein | 2.9E+08 |  |  |  |  |  |  |  |
| T0UVM5 | [head-tail connector]Uncharacterized protein | 2.86E+08 |  |  |  |  |  |  |  |
| T0V5R4 | Glutamine synthetase | 2.81E+08 |  |  |  |  |  |  |  |
| T0URR4 | ATP synthase subunit b | 2.76E+08 |  |  |  |  |  |  |  |
| T0ULH7 | Translation initiation factor IF-2 | 2.75E+08 |  |  |  |  |  |  |  |
| T0URZ7 | Amino acid ABC transporter permease | 2.71E+08 |  |  |  |  |  |  |  |
| T0UH11 | 50S ribosomal protein L13 | 2.67E+08 |  |  |  |  |  |  |  |
| T0ULX2 | Nitrogen regulatory protein P-II | 2.63E+08 |  |  |  |  |  |  |  |
| T0VMA2;T0UKD9 | Peptide ABC transporter ATPase | 2.54E+08 |  |  |  |  |  |  |  |
| T0UC37 | Serine hydroxymethyltransferase | 2.5E+08 |  |  |  |  |  |  |  |
| T0UT82 | Large-conductance mechanosensitive channel | 2.33E+08 |  |  |  |  |  |  |  |
| T0VSD6 | Flotillin | 2.29E+08 |  |  |  |  |  |  |  |
| T0VRF8 | Uncharacterized protein | 2.27E+08 |  |  |  |  |  |  |  |
| T0UK13 | Uncharacterized protein | 2.24E+08 |  |  |  |  |  |  |  |
| T0V5G0 | Crp/Fnr family transcriptional regulator | 2.17E+08 |  |  |  |  |  |  |  |
| T0UZ67 | 50S ribosomal protein L15 | 2.12E+08 |  |  |  |  |  |  |  |
| T0UIJ0 | DEAD-box ATP-dependent RNA helicase CshA | 2.11E+08 |  |  |  |  |  |  |  |
| T0WF61 | Septation ring formation regulator EzrA | 1.98E+08 |  |  |  |  |  |  |  |
| T0UYF1 | NADH dehydrogenase | 1.97E+08 |  |  |  |  |  |  |  |
| T0UYM6 | DNA-directed RNA polymerase subunit alpha | 1.96E+08 |  |  |  |  |  |  |  |
| T0VVB4 | GntR family transcriptional regulator | 1.94E+08 |  |  |  |  |  |  |  |
| T0VQ31 | Uncharacterized protein | 1.91E+08 |  |  |  |  |  |  |  |
| T0UPB9 | Tyrosine--tRNA ligase | 1.89E+08 |  |  |  |  |  |  |  |
| T0VTZ2 | dTDP-glucose 4,6-dehydratase | 1.86E+08 |  |  |  |  |  |  |  |
| T0UA32;T0UCB7 | Alpha-acetolactate decarboxylase | 1.82E+08 |  |  |  |  |  |  |  |
| T0UV91 | 50S ribosomal protein L10 | 1.77E+08 |  |  |  |  |  |  |  |
| T0USW8 | DNA primase | 1.76E+08 |  |  |  |  |  |  |  |
| T0UVT0 | Ribonucleoside-diphosphate reductase | 1.71E+08 |  |  |  |  |  |  |  |
| T0UQJ7 | Amino acid ABC transporter substrate-binding protein | 1.67E+08 |  |  |  |  |  |  |  |
| T0UKC2 | Cell division protein FtsZ | 1.65E+08 |  |  |  |  |  |  |  |
| T0UMT9 | Uncharacterized protein | 1.64E+08 |  |  |  |  |  |  |  |
| T0VYQ0 | EIIAB-Man | 1.63E+08 |  |  |  |  |  |  |  |
| T0UQ31 | Fumarate reductase | 1.61E+08 |  |  |  |  |  |  |  |
| T0VWP4 | 30S ribosomal protein S21 | 1.59E+08 |  |  |  |  |  |  |  |
| T0W625 | Uncharacterized protein | 1.59E+08 |  |  |  |  |  |  |  |
| T0UTJ0 | Proline--tRNA ligase | 1.57E+08 |  |  |  |  |  |  |  |
| T0W635 | 50S ribosomal protein L23 | 1.57E+08 |  |  |  |  |  |  |  |
| T0UT09 | Phosphoribosylaminoimidazole-succinocarboxamide synthase | 1.56E+08 |  |  |  |  |  |  |  |
| T0VX48;T0VSK8 | Site-specific DNA-methyltransferase (adenine-specific) (Fragment) | 1.55E+08 |  |  |  |  |  |  |  |
| T0UQF2 | Cell division protein FtsX | 1.52E+08 |  |  |  |  |  |  |  |
| T0WD50 | DUF536 domain-containing protein | 1.52E+08 |  |  |  |  |  |  |  |
| T0VT45 | Enolase | 1.51E+08 |  |  |  |  |  |  |  |
| T0UDZ9 | Uncharacterized protein | 1.49E+08 |  |  |  |  |  |  |  |
| T0VYA5 | ATP synthase gamma chain | 1.48E+08 |  |  |  |  |  |  |  |
| T0VW51 | Lysine--tRNA ligase | 1.47E+08 |  |  |  |  |  |  |  |
| T0UZ49 | Preprotein translocase subunit SecE | 1.47E+08 |  |  |  |  |  |  |  |
| T0UT31 | UDP-N-acetylmuramate--L-alanine ligase | 1.47E+08 |  |  |  |  |  |  |  |
| T0VXY4 | Aspartyl/glutamyl-tRNA(Asn/Gln) amidotransferase subunit B | 1.43E+08 |  |  |  |  |  |  |  |
| T0UPR3 | Lipoprotein | 1.42E+08 |  |  |  |  |  |  |  |
| T0V5I9 | 30S ribosomal protein S18 | 1.41E+08 |  |  |  |  |  |  |  |
| T0UL96 | Biotin carboxylase | 1.39E+08 |  |  |  |  |  |  |  |
| T0UW75 | Tail protein | 1.39E+08 |  |  |  |  |  |  |  |
| T0W628 | Uncharacterized protein | 1.38E+08 |  |  |  |  |  |  |  |
| T0UI20 | 2-oxoisovalerate dehydrogenase subunit beta | 1.37E+08 |  |  |  |  |  |  |  |
| T0UJS0 | Uncharacterized protein | 1.35E+08 |  |  |  |  |  |  |  |
| T0W8T6 | 50S ribosomal protein L18 | 1.35E+08 |  |  |  |  |  |  |  |
| T0UPM3 | Asparagine synthase (glutamine-hydrolyzing) | 1.35E+08 |  |  |  |  |  |  |  |
| T0UK73 | BRO-like protein (antirepressor) | 1.31E+08 |  |  |  |  |  |  |  |
| T0W1R9 | DNA-directed RNA polymerase subunit beta | 1.28E+08 |  |  |  |  |  |  |  |
| T0US03 | ATP synthase subunit delta | 1.28E+08 |  |  |  |  |  |  |  |
| T0USW5 | Uncharacterized protein | 1.27E+08 |  |  |  |  |  |  |  |
| T0W1M0 | Cell division protein SepF | 1.25E+08 |  |  |  |  |  |  |  |
| T0UBX9 | Endolytic murein transglycosylase | 1.24E+08 |  |  |  |  |  |  |  |
| T0VVI3 | [mobile element protein] Uncharacterized protein | 1.24E+08 |  |  |  |  |  |  |  |
| T0W8W3 | Phenylalanine--tRNA ligase alpha subunit | 1.23E+08 |  |  |  |  |  |  |  |
| T0V5G9 | Peptidase C51 domain-containing protein | 1.23E+08 |  |  |  |  |  |  |  |
| T0VZW4 | Protein RecA | 1.23E+08 |  |  |  |  |  |  |  |
| T0UBZ3 | General stress protein | 1.21E+08 |  |  |  |  |  |  |  |
| T0UYA6 | Histidine--tRNA ligase | 1.19E+08 |  |  |  |  |  |  |  |
| T0USU2 | Adenylosuccinate synthetase | 1.19E+08 |  |  |  |  |  |  |  |
| T0UDT1 | SHOCT domain-containing protein | 1.19E+08 |  |  |  |  |  |  |  |
| T0UMT0 | Glucose-1-phosphate thymidylyltransferase | 1.17E+08 |  |  |  |  |  |  |  |
| T0UP82 | DNA-binding protein HU | 1.17E+08 |  |  |  |  |  |  |  |
| T0UQB8 | Phage_Mu_F domain-containing protein | 1.16E+08 |  |  |  |  |  |  |  |
| T0UKD2 | Asparagine--tRNA ligase | 1.14E+08 |  |  |  |  |  |  |  |
| T0VVB1 | Phospho-2-dehydro-3-deoxyheptonate aldolase | 1.14E+08 |  |  |  |  |  |  |  |
| T0UHX1 | Pyruvate carboxylase | 1.14E+08 |  |  |  |  |  |  |  |
| T0VW85 | Peptidylprolyl isomerase | 1.13E+08 |  |  |  |  |  |  |  |
| T0W5P7 | 50S ribosomal protein L7/L12 | 1.13E+08 |  |  |  |  |  |  |  |
| T0V5Q2 | Chaperone protein DnaJ | 1.12E+08 |  |  |  |  |  |  |  |
| T0VR16 | Exopolysaccharide biosynthesis protein | 1.12E+08 |  |  |  |  |  |  |  |
| T0W8Q6 | ABC transporter substrate-binding protein | 1.11E+08 |  |  |  |  |  |  |  |
| T0UZA0 | Threonine--tRNA ligase | 1.11E+08 |  |  |  |  |  |  |  |
| T0UR35;T0UM78 | Foldase protein PrsA | 1.09E+08 |  |  |  |  |  |  |  |
| T0UHW1 | Ribosome hibernation promoting factor | 1.09E+08 |  |  |  |  |  |  |  |
| T0UT00 | Uncharacterized protein | 1.08E+08 |  |  |  |  |  |  |  |
| T0UZF9 | Glyceraldehyde-3-phosphate dehydrogenase | 1.06E+08 |  |  |  |  |  |  |  |
| T0VR37 | LytR family transcriptional regulator | 1.06E+08 |  |  |  |  |  |  |  |
| T0UIP9 | Ferrichrome ABC transporter substrate-binding protein | 1.05E+08 |  |  |  |  |  |  |  |
| T0US50 | Phosphate ABC transporter substrate-binding protein | 1.04E+08 |  |  |  |  |  |  |  |
| T0UVU3 | Holin | 1.04E+08 |  |  |  |  |  |  |  |
| T0W060 | Ribonucleoside triphosphate reductase | 1.04E+08 |  |  |  |  |  |  |  |
| T0UP19 | Heme ABC transporter ATP-binding protein | 1.03E+08 |  |  |  |  |  |  |  |
| T0W6H8 | Ribonuclease Y | 1.02E+08 |  |  |  |  |  |  |  |
| T0VP90 | Membrane protein insertase YidC | 1.01E+08 |  |  |  |  |  |  |  |
| T0W1N8 | Translation initiation factor IF-3 | 1.01E+08 |  |  |  |  |  |  |  |
| T0UK77 | Histidine kinase | 99177000 |  |  |  |  |  |  |  |
| T0USY8 | GTPase Obg | 96919500 |  |  |  |  |  |  |  |
| T0VXX9 | Glycosyltransferase | 96913000 |  |  |  |  |  |  |  |
| T0VW56 | Gamma-aminobutyrate permease | 96398500 |  |  |  |  |  |  |  |
| T0W6L5 | Uncharacterized protein | 96312000 |  |  |  |  |  |  |  |
| T0UAZ8 | Elongation factor G | 96160500 |  |  |  |  |  |  |  |
| T0UNP6 | Tellurite resistance protein TelA | 95810500 |  |  |  |  |  |  |  |
| T0UJ77 | Ribosome-binding ATPase YchF | 94270500 |  |  |  |  |  |  |  |
| T0UPK8 | 2,3-bisphosphoglycerate-dependent phosphoglycerate mutase | 91378500 |  |  |  |  |  |  |  |
| T0W359 | NADH dehydrogenase | 90689000 |  |  |  |  |  |  |  |
| T0UK98 | Cell division protein FtsZ (Fragment) | 90191000 |  |  |  |  |  |  |  |
| T0W2R5 | Cell division ATP-binding protein FtsE | 90062000 |  |  |  |  |  |  |  |
| T0VZ42 | Protein translocase subunit SecA | 89197500 |  |  |  |  |  |  |  |
| T0URK2 | DNA-directed RNA polymerase subunit beta | 89058500 |  |  |  |  |  |  |  |
| T0UER7 | Transcriptional repressor NrdR | 88485650 |  |  |  |  |  |  |  |
| T0VVD6 | Short-chain dehydrogenase | 88236500 |  |  |  |  |  |  |  |
| T0USZ0 | Ammonium transporter | 86084000 |  |  |  |  |  |  |  |
| T0V5H3 | HAD family hydrolase | 85857700 |  |  |  |  |  |  |  |
| T0UTB1 | 30S ribosomal protein S14 type Z | 85536000 |  |  |  |  |  |  |  |
| T0UW92 | Redox-sensing transcriptional repressor Rex | 84880500 |  |  |  |  |  |  |  |
| T0VVD0 | [transcriptional regulation protein] Uncharacterized protein | 84427500 |  |  |  |  |  |  |  |
| T0W0B1 | GTPase | 84245500 |  |  |  |  |  |  |  |
| T0UPT1 | GTPase Era | 84055500 |  |  |  |  |  |  |  |
| T0UK07 | Uncharacterized protein | 83717500 |  |  |  |  |  |  |  |
| T0UDY2 | Methyltransferase | 83416500 |  |  |  |  |  |  |  |
| T0W1P9 | Pyrophosphate phospho-hydrolase | 82597050 |  |  |  |  |  |  |  |
| T0W680 | Uracil phosphoribosyltransferase | 82039600 |  |  |  |  |  |  |  |
| T0UPZ4 | UDP-N-acetylglucosamine 1-carboxyvinyltransferase | 80494500 |  |  |  |  |  |  |  |
| T0UQ28 | Inosine-5-monophosphate dehydrogenase | 79515500 |  |  |  |  |  |  |  |
| T0UYS8 | Uridylate kinase | 79507500 |  |  |  |  |  |  |  |
| T0VU22 | Uncharacterized protein | 78692350 |  |  |  |  |  |  |  |
| T0UES5 | Aspartokinase | 78412500 |  |  |  |  |  |  |  |
| T0UKX8 | tRNA (guanine-N(7)-)-methyltransferase | 78076000 |  |  |  |  |  |  |  |
| T0UPC1 | Pyruvate dehydrogenase E1 component subunit alpha | 77815000 |  |  |  |  |  |  |  |
| T0UQT9 | DNA-directed RNA polymerase subunit omega | 77753000 |  |  |  |  |  |  |  |
| T0VRF3 | Uncharacterized protein | 76442000 |  |  |  |  |  |  |  |
| T0UZV7 | Site-specific DNA-methyltransferase (adenine-specific) (Fragment) | 75885500 |  |  |  |  |  |  |  |
| T0UMX9 | Membrane protein insertase YidC | 74790000 |  |  |  |  |  |  |  |
| T0UYL4 | 50S ribosomal protein L22 | 74064260 |  |  |  |  |  |  |  |
| T0UVI1 | Glycine--tRNA ligase alpha subunit | 73207500 |  |  |  |  |  |  |  |
| T0VZC9 | Thymidylate synthase | 72678000 |  |  |  |  |  |  |  |
| T0W8H7 | tRNA-dihydrouridine synthase | 72651000 |  |  |  |  |  |  |  |
| T0UTU9 | Uncharacterized protein | 71466600 |  |  |  |  |  |  |  |
| T0UPY7 | L-lactate dehydrogenase | 71454000 |  |  |  |  |  |  |  |
| T0URV9 | HAD hydrolase | 70586250 |  |  |  |  |  |  |  |
| T0UKY5 | Chorismate synthase | 69618000 |  |  |  |  |  |  |  |
| T0UM65 | P-type Ca(2+) transporter | 69543500 |  |  |  |  |  |  |  |
| T0UFR2 | Tagatose 1,6-diphosphate aldolase | 69432500 |  |  |  |  |  |  |  |
| T0W4S5 | Heme ABC transporter ATP-binding protein | 69039500 |  |  |  |  |  |  |  |
| T0W2M8 | Signal recognition particle protein | 68820650 |  |  |  |  |  |  |  |
| T0UVB9 | Uncharacterized protein | 68756500 |  |  |  |  |  |  |  |
| T0UVA7;T0UR10 | Holin | 68505000 |  |  |  |  |  |  |  |
| T0UW47;T0UI22 | Sugar phosphate phosphatase | 68105000 |  |  |  |  |  |  |  |
| T0UHK2 | Uncharacterized protein | 67600500 |  |  |  |  |  |  |  |
| T0VU18 | Glycosyl transferase | 67484000 |  |  |  |  |  |  |  |
| T0UPJ1 | DEAD-box ATP-dependent RNA helicase CshB | 67343500 |  |  |  |  |  |  |  |
| T0W379 | Carbamoyl-phosphate synthase large chain | 66892500 |  |  |  |  |  |  |  |
| T0W5V2 | Niacin transporter NiaX | 66149850 |  |  |  |  |  |  |  |
| T0URR1 | ATP synthase epsilon chain | 65427000 |  |  |  |  |  |  |  |
| T0VTV5 | Aminotransferase AlaT | 65200500 |  |  |  |  |  |  |  |
| T0VTW5 | Uncharacterized protein | 64529400 |  |  |  |  |  |  |  |
| T0WD54 | Uncharacterized protein | 63934500 |  |  |  |  |  |  |  |
| T0UVE1 | Homoserine kinase | 63921915 |  |  |  |  |  |  |  |
| T0UHB9 | ATP-dependent Clp protease ATP-binding protein | 63783000 |  |  |  |  |  |  |  |
| T0UER5 | GTPase Der | 62793500 |  |  |  |  |  |  |  |
| T0UK87 | [DNA binding protein]Uncharacterized protein | 62480650 |  |  |  |  |  |  |  |
| T0W5X7 | FAD:protein FMN transferase | 62022400 |  |  |  |  |  |  |  |
| T0UNX4 | (p)ppGpp synthase | 61826000 |  |  |  |  |  |  |  |
| T0W679 | Protein GrpE | 61610000 |  |  |  |  |  |  |  |
| T0UQ10 | Spermidine/putrescine import ATP-binding protein PotA | 61380500 |  |  |  |  |  |  |  |
| T0USN1 | Carbamoyl-phosphate synthase small chain | 61108000 |  |  |  |  |  |  |  |
| T0UVB2 | [receptor binding protein]Uncharacterized protein | 60767350 |  |  |  |  |  |  |  |
| T0UIK8 | ABC transporter ATPase | 60445600 |  |  |  |  |  |  |  |
| T0UKD4 | Uncharacterized protein | 60371000 |  |  |  |  |  |  |  |
| T0UQS6 | Fructose-bisphosphate aldolase | 60329500 |  |  |  |  |  |  |  |
| T0UJY2 | dUTP diphosphatase | 60233000 |  |  |  |  |  |  |  |
| T0VVK9 | Uncharacterized protein | 59810000 |  |  |  |  |  |  |  |
| T0W2A3;T0VXJ2;T0ULM6 | 1,4-beta-N-acetylmuramidase (Fragment) | 59445850 |  |  |  |  |  |  |  |
| T0UJ83 | Hypoxanthine phosphoribosyltransferase | 59352900 |  |  |  |  |  |  |  |
| T0VVJ0 | UDP-N-acetylglucosamine 1-carboxyvinyltransferase | 59332500 |  |  |  |  |  |  |  |
| T0UZI3 | Uncharacterized protein | 59084000 |  |  |  |  |  |  |  |
| T0UYM4 | Protein translocase subunit SecY | 59080000 |  |  |  |  |  |  |  |
| T0VXU3 | Bifunctional protein GlmU | 57849100 |  |  |  |  |  |  |  |
| T0ULH3 | 3-hydroxyacyl-[acyl-carrier-protein] dehydratase FabZ | 57158000 |  |  |  |  |  |  |  |
| T0UQC2 | LACLC[capsid and scaffolding protein] Uncharacterized protein | 57077950 |  |  |  |  |  |  |  |
| T0W2G0 | ATP-dependent DNA helicase | 56707500 |  |  |  |  |  |  |  |
| T0VYZ3 | Bifunctional protein PyrR | 56666000 |  |  |  |  |  |  |  |
| T0W5V6 | Spermidine/putrescine ABC transporter substrate-binding protein | 56265000 |  |  |  |  |  |  |  |
| T0UMN7 | Glutamyl-tRNA(Gln) amidotransferase subunit A | 56036000 |  |  |  |  |  |  |  |
| T0W2P9 | Glutamine--fructose-6-phosphate aminotransferase [isomerizing] | 55745800 |  |  |  |  |  |  |  |
| T0URB6 | Cell division protein FtsA | 55595050 |  |  |  |  |  |  |  |
| T0UGK4 | Pur operon repressor | 54386500 |  |  |  |  |  |  |  |
| T0W673 | Phenylalanine--tRNA ligase beta subunit | 53763950 |  |  |  |  |  |  |  |
| T0VXX8 | UvrABC system protein A | 53728500 |  |  |  |  |  |  |  |
| T0WFB9 | Asparagine synthase | 53324900 |  |  |  |  |  |  |  |
| T0UQ33 | 2,3,4,5-tetrahydropyridine-2,6-dicarboxylate N-acetyltransferase | 52994150 |  |  |  |  |  |  |  |
| T0W2I2 | Mid-cell-anchored protein Z | 52317450 |  |  |  |  |  |  |  |
| T0VXW2 | Universal stress protein | 52215795 |  |  |  |  |  |  |  |
| T0URU6 | Phosphate import ATP-binding protein PstB | 52112900 |  |  |  |  |  |  |  |
| T0W8H5 | Aspartate--tRNA ligase | 51642750 |  |  |  |  |  |  |  |
| T0VV94 | 50S ribosomal protein L33 | 51281000 |  |  |  |  |  |  |  |
| T0VZG8 | Uncharacterized protein | 51065500 |  |  |  |  |  |  |  |
| T0W225 | Phosphate import ATP-binding protein PstB | 50084500 |  |  |  |  |  |  |  |
| T0UV12 | TPR_REGION domain-containing protein | 49008800 |  |  |  |  |  |  |  |
| T0W6A8 | Zinc metalloprotease | 48960500 |  |  |  |  |  |  |  |
| T0UUJ7 | Glycerol-3-phosphate dehydrogenase [NAD(P)+] | 48665050 |  |  |  |  |  |  |  |
| T0WF59 | MFS transporter | 48654230 |  |  |  |  |  |  |  |
| T0UPX4 | [upper base plate protein]Uncharacterized protein | 48337500 |  |  |  |  |  |  |  |
| T0UKB9 | Cell division protein DivIVA | 48094000 |  |  |  |  |  |  |  |
| T0UR70 | Non-specific serine/threonine protein kinase | 47429500 |  |  |  |  |  |  |  |
| T0W6E3 | 3-dehydroquinate dehydratase | 47422400 |  |  |  |  |  |  |  |
| T0UZH2 | Cell shape-determining protein MreC | 47414950 |  |  |  |  |  |  |  |
| T0W2C9 | Probable GTP-binding protein EngB | 47141850 |  |  |  |  |  |  |  |
| T0UMQ9 | Major cold shock protein | 46264500 |  |  |  |  |  |  |  |
| T0UY72 | DNA methyltransferase | 46165150 |  |  |  |  |  |  |  |
| T0USA3 | Dipeptidase | 46071400 |  |  |  |  |  |  |  |
| T0W2L7 | Uracil transporter | 45965000 |  |  |  |  |  |  |  |
| T0UPH9;T0UGS1 | 60 kDa chaperonin | 45949500 |  |  |  |  |  |  |  |
| T0UJR5 | Cation transporter | 45948800 |  |  |  |  |  |  |  |
| T0VZ02 | Ribonuclease J | 45806500 |  |  |  |  |  |  |  |
| T0URS8 | DNA repair protein RecN | 45804400 |  |  |  |  |  |  |  |
| T0W1X2 | ATP synthase subunit a | 45698500 |  |  |  |  |  |  |  |
| T0VZD8 | Ribose-phosphate pyrophosphokinase | 45338150 |  |  |  |  |  |  |  |
| T0UYY6 | Uncharacterized protein | 45272000 |  |  |  |  |  |  |  |
| T0UPS4 | XRE family transcriptional regulator | 45206000 |  |  |  |  |  |  |  |
| T0W5U4 | ATP-dependent Clp protease ATP-binding subunit ClpX | 44886050 |  |  |  |  |  |  |  |
| T0UPX0 | Energy-coupling factor transporter ATP-binding protein EcfA | 44846935 |  |  |  |  |  |  |  |
| T0UPJ8 | Pyruvate-flavodoxin oxidoreductase | 44730950 |  |  |  |  |  |  |  |
| T0VTU6 | RNA polymerase sigma factor SigA | 44726850 |  |  |  |  |  |  |  |
| T0UJ20 | Uncharacterized protein (Fragment) | 44481500 |  |  |  |  |  |  |  |
| T0W2A0 | [tail element protein] Uncharacterized protein | 44059050 |  |  |  |  |  |  |  |
| T0VP60 | 2-oxoglutarate reductase | 44026800 |  |  |  |  |  |  |  |
| T0UVK6 | Methylenetetrahydrofolate--tRNA-(uracil-5-)-methyltransferase TrmFO | 43866900 |  |  |  |  |  |  |  |
| T0VWJ1 | Ribonuclease J | 43653450 |  |  |  |  |  |  |  |
| T0UKD7 | Universal stress protein UspA | 43595400 |  |  |  |  |  |  |  |
| T0VZA2 | UDP-N-acetylmuramoylalanine--D-glutamate ligase | 43554500 |  |  |  |  |  |  |  |
| T0UPB6 | Probable tRNA sulfurtransferase | 43285150 |  |  |  |  |  |  |  |
| T0W8E0 | Acetate kinase | 43212850 |  |  |  |  |  |  |  |
| T0VWV0 | Hydrolase | 42989650 |  |  |  |  |  |  |  |
| T0VT73 | Probable potassium transport system protein kup | 42928250 |  |  |  |  |  |  |  |
| T0UGP8 | UDP-galactopuranose mutase | 42857000 |  |  |  |  |  |  |  |
| T0UVU9 | Heat-inducible transcription repressor HrcA | 42637000 |  |  |  |  |  |  |  |
| T0ULR8 | Dihydroorotate dehydrogenase | 42252250 |  |  |  |  |  |  |  |
| T0UPW1 | Ribosome biogenesis GTPase A | 42072650 |  |  |  |  |  |  |  |
| T0UKY4 | Cyclic-di-AMP phosphodiesterase | 41823400 |  |  |  |  |  |  |  |
| T0W8F7 | HTH_11 domain-containing protein | 41275000 |  |  |  |  |  |  |  |
| T0WD64 | Carboxylate--amine ligase | 41129350 |  |  |  |  |  |  |  |
| T0UJY6 | DNA replication protein | 40892050 |  |  |  |  |  |  |  |
| T0UHY3 | Uncharacterized protein | 40840500 |  |  |  |  |  |  |  |
| T0UMU6 | Glycos_transf_1 domain-containing protein | 40144950 |  |  |  |  |  |  |  |
| T0UYT2 | 1,2-diacylglycerol 3-glucosyltransferase | 39767100 |  |  |  |  |  |  |  |
| T0W078 | Cation transporter | 39416000 |  |  |  |  |  |  |  |
| T0VZI2 | FMN-dependent NADH-azoreductase | 39284600 |  |  |  |  |  |  |  |
| T0VZ56 | Uncharacterized protein | 38925500 |  |  |  |  |  |  |  |
| T0WF88 | Endopeptidase La | 38576050 |  |  |  |  |  |  |  |
| T0UMR0 | Dihydroxyacetone kinase | 38515000 |  |  |  |  |  |  |  |
| T0UGL7 | Uncharacterized protein | 37893250 |  |  |  |  |  |  |  |
| T0UUX1 | 5-bromo-4-chloroindolyl phosphate hydrolysis protein | 37836100 |  |  |  |  |  |  |  |
| T0VWX6 | N5-carboxyaminoimidazole ribonucleotide synthase | 37816800 |  |  |  |  |  |  |  |
| T0UVW7 | UPF0145 protein LLT1_7225 | 37722500 |  |  |  |  |  |  |  |
| T0VW65 | NADH dehydrogenase | 36004150 |  |  |  |  |  |  |  |
| T0UPA1 | LytR family transcriptional regulator | 35904800 |  |  |  |  |  |  |  |
| T0V5E6 | Uncharacterized protein | 35721950 |  |  |  |  |  |  |  |
| T0UPW8 | Peptidoglycan hydrolase | 35060000 |  |  |  |  |  |  |  |
| T0UCQ3 | Glycine/betaine ABC transporter permease | 34974000 |  |  |  |  |  |  |  |
| T0WFD0 | Glucose-6-phosphate 1-dehydrogenase | 34969250 |  |  |  |  |  |  |  |
| T0UP98 | Trigger factor | 34848000 |  |  |  |  |  |  |  |
| T0VSV9 | Bifunctional purine biosynthesis protein PurH | 34104500 |  |  |  |  |  |  |  |
| T0UKG2 | Uncharacterized protein | 34055050 |  |  |  |  |  |  |  |
| T0UVP2 | Pseudouridine synthase | 34027800 |  |  |  |  |  |  |  |
| T0W2N0 | Terminase | 33803050 |  |  |  |  |  |  |  |
| T0USR6 | 30S ribosomal protein S16 | 33650200 |  |  |  |  |  |  |  |
| T0VZ39 | Uncharacterized protein | 33556000 |  |  |  |  |  |  |  |
| T0W5R2 | Protease | 33373950 |  |  |  |  |  |  |  |
| T0UID7 | Uncharacterized protein | 33273500 |  |  |  |  |  |  |  |
| T0VWV5 | Chromosomal replication initiator protein DnaA | 33187050 |  |  |  |  |  |  |  |
| T0ULA0 | Malonyl CoA-acyl carrier protein transacylase | 33179400 |  |  |  |  |  |  |  |
| T0VYI4 | 3-phosphoshikimate 1-carboxyvinyltransferase | 33128150 |  |  |  |  |  |  |  |
| T0UYL8 | 50S ribosomal protein L14 | 33064200 |  |  |  |  |  |  |  |
| T0UI46 | Isochorismatase | 32554050 |  |  |  |  |  |  |  |
| T0UPR8 | Methionine import ATP-binding protein MetN | 32359900 |  |  |  |  |  |  |  |
| T0W2W9 | UDP-N-acetylglucosamine--N-acetylmuramyl-(pentapeptide) pyrophosphoryl-undecaprenol N-acetylglucosamine transferase | 32238050 |  |  |  |  |  |  |  |
| T0VRD3 | Crossover junction endodeoxyribonuclease RusA | 32129000 |  |  |  |  |  |  |  |
| T0UQE0 | Dihydroorotase | 32067650 |  |  |  |  |  |  |  |
| T0US48 | Peptidoglycan-binding protein LysM | 31964850 |  |  |  |  |  |  |  |
| T0W2B1 | Riboflavin biosynthesis protein | 31883650 |  |  |  |  |  |  |  |
| T0VY29 | Uncharacterized protein | 31365950 |  |  |  |  |  |  |  |
| T0UMV4 | Glycosyl transferase | 31351100 |  |  |  |  |  |  |  |
| T0W5Z2 | Glycine--tRNA ligase beta subunit | 30914550 |  |  |  |  |  |  |  |
| T0VTZ7 | ABC transporter ATP-binding protein | 30853450 |  |  |  |  |  |  |  |
| T0UGP3 | Lipopolysaccharide biosynthesis protein | 30701250 |  |  |  |  |  |  |  |
| T0UQ32 | Probable nicotinate-nucleotide adenylyltransferase | 30553700 |  |  |  |  |  |  |  |
| T0UKL3 | Ribosomal RNA small subunit methyltransferase A | 30406600 |  |  |  |  |  |  |  |
| T0UZA7 | Zinc ABC transporter substrate-binding protein | 30332150 |  |  |  |  |  |  |  |
| T0UFD1 | Hypoxanthine phosphoribosyltransferase | 30185950 |  |  |  |  |  |  |  |
| T0UYX4 | ABC transporter ATP-binding protein | 30037500 |  |  |  |  |  |  |  |
| T0UPD4 | Ribosomal RNA small subunit methyltransferase I | 29764750 |  |  |  |  |  |  |  |
| T0UNQ7 | Dihydroorotate dehydrogenase B (NAD(+)), electron transfer subunit | 29722750 |  |  |  |  |  |  |  |
| T0UT77 | Acyltransferase | 29244300 |  |  |  |  |  |  |  |
| T0VXZ6 | Uncharacterized protein | 29046000 |  |  |  |  |  |  |  |
| T0UVU7 | Uncharacterized protein | 29025750 |  |  |  |  |  |  |  |
| T0UZE5 | RNA methyltransferase | 28826000 |  |  |  |  |  |  |  |
| T0UVN6 | HeH domain-containing protein | 28769150 |  |  |  |  |  |  |  |
| T0UPI7 | Diphosphomevalonate decarboxylase | 28756750 |  |  |  |  |  |  |  |
| T0VZA0 | DD-transpeptidase | 28753750 |  |  |  |  |  |  |  |
| T0VZV2 | UDP-N-acetylmuramoyl-tripeptide--D-alanyl-D-alanine ligase | 28691300 |  |  |  |  |  |  |  |
| T0UUJ2 | Pyruvate kinase | 28617650 |  |  |  |  |  |  |  |
| T0UL57 | Serine--tRNA ligase | 28508650 |  |  |  |  |  |  |  |
| T0W6A3 | Serine protease | 28177850 |  |  |  |  |  |  |  |
| T0US52 | Regulator | 27538450 |  |  |  |  |  |  |  |
| T0UZ56 | 30S ribosomal protein S19 | 27496200 |  |  |  |  |  |  |  |
| T0UVR2 | Pseudouridine synthase | 27390000 |  |  |  |  |  |  |  |
| T0W2G9 | Sortase | 27384450 |  |  |  |  |  |  |  |
| T0UBU6 | Acetate--CoA ligase | 27254850 |  |  |  |  |  |  |  |
| T0W2B5 | Triosephosphate isomerase | 26624900 |  |  |  |  |  |  |  |
| T0W5U0 | Repressor | 26574500 |  |  |  |  |  |  |  |
| T0UVI5 | UPF0176 protein LLT1_10055 | 26241350 |  |  |  |  |  |  |  |
| T0UP24 | Polysaccharide deacetylase | 26126300 |  |  |  |  |  |  |  |
| T0UKM0 | Sodium ABC transporter ATP-binding protein | 25782300 |  |  |  |  |  |  |  |
| T0VZC1 | Phosphoribosylformylglycinamidine synthase subunit PurQ | 25555000 |  |  |  |  |  |  |  |
| T0USB7 | Uridine kinase | 25477300 |  |  |  |  |  |  |  |
| T0VTX5 | Probable dual-specificity RNA methyltransferase RlmN | 25339450 |  |  |  |  |  |  |  |
| T0UP92 | Diadenylate cyclase | 25317650 |  |  |  |  |  |  |  |
| T0UFS7 | DeoR family transcriptional regulator | 25116150 |  |  |  |  |  |  |  |
| T0W676 | 3-5 exoribonuclease | 25017405 |  |  |  |  |  |  |  |
| T0W065 | Energy-coupling factor transporter ATP-binding protein EcfA | 24982650 |  |  |  |  |  |  |  |
| T0UQW8;T0W381 | [CAAX protease]Uncharacterized protein | 24880250 |  |  |  |  |  |  |  |
| T0UQ86 | Uncharacterized protein | 24855900 |  |  |  |  |  |  |  |
| T0URX0 | DeoR family transcriptional regulator | 24734600 |  |  |  |  |  |  |  |
| T0W2H6 | Oxidoreductase | 24709500 |  |  |  |  |  |  |  |
| T0VXV3 | rRNA methyltransferase | 24607650 |  |  |  |  |  |  |  |
| T0UTG5 | Ribonucleotide reductase | 24540900 |  |  |  |  |  |  |  |
| T0VWE5 | Uncharacterized protein | 24521500 |  |  |  |  |  |  |  |
| T0UMU9 | Uncharacterized protein (Fragment) | 24509950 |  |  |  |  |  |  |  |
| T0W604 | Twitching motility protein pilT | 24183750 |  |  |  |  |  |  |  |
| T0VW64 | Transcription antitermination protein NusB | 24134050 |  |  |  |  |  |  |  |
| T0VZI7 | DD-transpeptidase | 24039500 |  |  |  |  |  |  |  |
| T0UT30 | L-serine dehydratase | 23999600 |  |  |  |  |  |  |  |
| T0VW76 | Amino acid transporter | 23926750 |  |  |  |  |  |  |  |
| T0VSC8 | Oligoribonuclease | 23864750 |  |  |  |  |  |  |  |
| T0W2Y8 | Exodeoxyribonuclease 7 large subunit | 23843200 |  |  |  |  |  |  |  |
| T0UVX5 | Cobalt ABC transporter ATP-binding protein | 23787300 |  |  |  |  |  |  |  |
| T0UKE3 | Uncharacterized protein | 23605850 |  |  |  |  |  |  |  |
| T0UQ37 | S1 RNA-binding protein | 23283450 |  |  |  |  |  |  |  |
| T0UIY9 | GTPase HflX | 22935500 |  |  |  |  |  |  |  |
| T0URS9 | Penicillin-binding protein 2X | 22802350 |  |  |  |  |  |  |  |
| T0VZ29 | Recombinase RarA | 22714150 |  |  |  |  |  |  |  |
| T0UHU3 | Ribosomal RNA small subunit methyltransferase E | 22503050 |  |  |  |  |  |  |  |
| T0UGG3 | Chromosome partitioning protein ParA | 22478750 |  |  |  |  |  |  |  |
| T0UP15 | Phosphate acyltransferase | 22442100 |  |  |  |  |  |  |  |
| T0W151 | Branched-chain amino acid ABC transporter permease | 22237400 |  |  |  |  |  |  |  |
| T0UVG4 | DNA gyrase subunit A | 22229450 |  |  |  |  |  |  |  |
| T0UR37 | Universal stress protein | 22180600 |  |  |  |  |  |  |  |
| T0W8X2 | RmuC family endonuclease | 22074550 |  |  |  |  |  |  |  |
| T0UPC5 | tRNA-binding protein | 21901500 |  |  |  |  |  |  |  |
| T0UYW3 | S4 RNA-binding domain-containing protein | 21888500 |  |  |  |  |  |  |  |
| T0ULN4 | GMP synthase [glutamine-hydrolyzing] | 21832250 |  |  |  |  |  |  |  |
| T0US54 | Alanine--tRNA ligase | 21643900 |  |  |  |  |  |  |  |
| T0UVV3 | Lactose transport regulator | 21635100 |  |  |  |  |  |  |  |
| T0UQP4 | DUF4767 domain-containing protein | 21473850 |  |  |  |  |  |  |  |
| T0W2I7 | NH(3)-dependent NAD(+) synthetase | 21433450 |  |  |  |  |  |  |  |
| T0W2L5 | Phosphate starvation-inducible protein PhoH | 21355750 |  |  |  |  |  |  |  |
| T0VYR5 | Demethylmenaquinone methyltransferase | 21264350 |  |  |  |  |  |  |  |
| T0UVS1 | Nucleotide-binding protein LLT1_7985 | 21242300 |  |  |  |  |  |  |  |
| T0US23 | Cell division protein FtsL | 21138450 |  |  |  |  |  |  |  |
| T0UPD5 | RNA methyltransferase | 21130500 |  |  |  |  |  |  |  |
| T0VW71 | Peptide chain release factor 3 | 21104550 |  |  |  |  |  |  |  |
| T0UT74 | tRNA-specific 2-thiouridylase MnmA | 20928250 |  |  |  |  |  |  |  |
| T0UGI3 | DNAse | 20901600 |  |  |  |  |  |  |  |
| T0URJ3 | Fe-S cluster assembly protein SufB | 20825700 |  |  |  |  |  |  |  |
| T0UGK9 | Nitroreductase | 20622650 |  |  |  |  |  |  |  |
| T0UKS1 | Ribosomal RNA small subunit methyltransferase H | 20580500 |  |  |  |  |  |  |  |
| T0W1K6 | Putative ribose-phosphate pyrophosphokinase | 20580150 |  |  |  |  |  |  |  |
| T0W5T9 | Xanthine phosphoribosyltransferase | 20507200 |  |  |  |  |  |  |  |
| T0UHU7 | 1-acyl-sn-glycerol-3-phosphate acyltransferase | 20422150 |  |  |  |  |  |  |  |
| T0USU7 | Bifunctional protein FolD | 20366400 |  |  |  |  |  |  |  |
| T0W6G4 | Hydrolase | 20359350 |  |  |  |  |  |  |  |
| T0VSW2 | Chaperone protein ClpB | 20257450 |  |  |  |  |  |  |  |
| T0VSL7 | Acetyl-CoA carboxytransferase | 20077650 |  |  |  |  |  |  |  |
| T0W4T8 | Orotidine 5-phosphate decarboxylase | 20031200 |  |  |  |  |  |  |  |
| T0W2P5 | Glycosyltransferase | 19813100 |  |  |  |  |  |  |  |
| T0W1Q1 | Macrolide ABC transporter ATP-binding protein | 19722300 |  |  |  |  |  |  |  |
| T0W338 | LuxR family transcriptional regulator | 19638250 |  |  |  |  |  |  |  |
| T0USY9 | Membrane protein | 19549215 |  |  |  |  |  |  |  |
| T0W4U7 | GntR family transcriptional regulator | 19471350 |  |  |  |  |  |  |  |
| T0VT54 | AAA_31 domain-containing protein | 19233800 |  |  |  |  |  |  |  |
| T0UIG0 | 3-hydroxyacyl-[acyl-carrier-protein] dehydratase FabZ | 19115500 |  |  |  |  |  |  |  |
| T0USY0 | Haloacid dehalogenase | 18963300 |  |  |  |  |  |  |  |
| T0UQU5 | Methionyl-tRNA formyltransferase | 18933800 |  |  |  |  |  |  |  |
| T0V5L7 | Valine--tRNA ligase | 18925400 |  |  |  |  |  |  |  |
| T0UVY6 | Homoserine dehydrogenase | 18669200 |  |  |  |  |  |  |  |
| T0UI54 | UvrABC system protein B | 18506050 |  |  |  |  |  |  |  |
| T0UYZ3;T0VR83 | Uncharacterized protein | 18378850 |  |  |  |  |  |  |  |
| T0VT82 | Adenine phosphoribosyltransferase | 18143175 |  |  |  |  |  |  |  |
| T0VS82 | Signal peptidase I | 18142800 |  |  |  |  |  |  |  |
| T0UL94 | Haloacid dehalogenase | 18051500 |  |  |  |  |  |  |  |
| T0UIZ2 | Ribosomal silencing factor RsfS | 18024500 |  |  |  |  |  |  |  |
| T0W1N2 | DUF5590 domain-containing protein | 17976350 |  |  |  |  |  |  |  |
| T0UGM6 | Glycosyl transferase family 2 | 17917350 |  |  |  |  |  |  |  |
| T0UII0 | Penicillin-binding protein 2B | 17866900 |  |  |  |  |  |  |  |
| T0VP67 | Sua5/YciO/YrdC/YwlC family RNA-binding protein | 17818200 |  |  |  |  |  |  |  |
| T0UI24 | Uncharacterized protein | 17789500 |  |  |  |  |  |  |  |
| T0VZG3 | tRNA pseudouridine synthase A | 17611600 |  |  |  |  |  |  |  |
| T0VRG0 | [DNA topoisomerase] Uncharacterized protein | 17500300 |  |  |  |  |  |  |  |
| T0UVX9 | GTP cyclohydrolase 1 | 17480250 |  |  |  |  |  |  |  |
| T0UQB2 | Uncharacterized protein | 17473000 |  |  |  |  |  |  |  |
| T0VVX9 | Uncharacterized protein | 17469950 |  |  |  |  |  |  |  |
| T0V5G3 | Chromosome partitioning protein ParB | 17266600 |  |  |  |  |  |  |  |
| T0W957 | Uncharacterized protein | 17266300 |  |  |  |  |  |  |  |
| T0UM12 | Uncharacterized protein | 17258250 |  |  |  |  |  |  |  |
| T0UL48 | Transcriptional regulator | 17240900 |  |  |  |  |  |  |  |
| T0VVA4 | Ribosomal protein L11 methyltransferase | 17172850 |  |  |  |  |  |  |  |
| T0UNJ2 | TPM_phosphatase domain-containing protein | 16959600 |  |  |  |  |  |  |  |
| T0UL90 | HAD family hydrolase | 16903050 |  |  |  |  |  |  |  |
| T0ULS1 | Membrane protein | 16882950 |  |  |  |  |  |  |  |
| T0UMX2 | RNA-binding protein | 16624850 |  |  |  |  |  |  |  |
| T0W0B7 | Probable transcriptional regulatory protein LLT1_5485 | 16590700 |  |  |  |  |  |  |  |
| T0UJ02 | Uracil-DNA glycosylase | 16367300 |  |  |  |  |  |  |  |
| T0UES9 | Uncharacterized protein | 16340650 |  |  |  |  |  |  |  |
| T0VYC5 | Transporter | 16211200 |  |  |  |  |  |  |  |
| T0W651 | DNA repair protein RadA | 16132900 |  |  |  |  |  |  |  |
| T0US59 | Guanine permease | 15993550 |  |  |  |  |  |  |  |
| T0VZL6 | DNA ligase | 15908600 |  |  |  |  |  |  |  |
| T0URS3 | Iron ABC transporter ATP-binding protein | 15876400 |  |  |  |  |  |  |  |
| T0UQF8 | Septum formation initiator | 15738400 |  |  |  |  |  |  |  |
| T0UW97 | Cardiolipin synthase | 15594250 |  |  |  |  |  |  |  |
| T0W4M9 | Virion core protein | 15531100 |  |  |  |  |  |  |  |
| T0VNY5 | Phosphatidylglycerol--prolipoprotein diacylglyceryl transferase | 15490000 |  |  |  |  |  |  |  |
| T0VLW1 | 6-phospho-beta-galactosidase | 15391000 |  |  |  |  |  |  |  |
| T0UFG6 | Phosphoribosylamine--glycine ligase | 15357550 |  |  |  |  |  |  |  |
| T0W160 | Dihydroorotate dehydrogenase | 15331940 |  |  |  |  |  |  |  |
| T0ULX3 | Anthranilate phosphoribosyltransferase | 15308250 |  |  |  |  |  |  |  |
| T0V5B1 | Aldehyde-alcohol dehydrogenase | 15122750 |  |  |  |  |  |  |  |
| T0W2C0 | Acyl-ACP thioesterase | 15075750 |  |  |  |  |  |  |  |
| T0UHH3 | Oxidoreductase | 15065100 |  |  |  |  |  |  |  |
| T0UW91;T0UI89;T0W148 | Aminoglycoside N(3)-acetyltransferase | 15058150 |  |  |  |  |  |  |  |
| T0W6B2 | DNA-directed DNA polymerase (Fragment) | 15057300 |  |  |  |  |  |  |  |
| T0W5U3 | Uncharacterized protein | 15020500 |  |  |  |  |  |  |  |
| T0W2U7 | Chaperone protein DnaK | 14941100 |  |  |  |  |  |  |  |
| T0W6F6 | Sensor histidine kinase | 14830350 |  |  |  |  |  |  |  |
| T0UPH5 | Thioredoxin | 14782860 |  |  |  |  |  |  |  |
| T0W074 | tRNA N6-adenosine threonylcarbamoyltransferase | 14752800 |  |  |  |  |  |  |  |
| T0W1R2 | Uncharacterized protein | 14702950 |  |  |  |  |  |  |  |
| T0UUW9 | L-lactate dehydrogenase | 14660700 |  |  |  |  |  |  |  |
| T0UVI4 | Nicotinate phosphoribosyltransferase | 14580300 |  |  |  |  |  |  |  |
| T0V5R1 | DNA mismatch repair protein MutL | 14577650 |  |  |  |  |  |  |  |
| T0W312 | SAM-dependent methyltransferase | 14576600 |  |  |  |  |  |  |  |
| T0USY4 | UPF0109 protein LLT1_11195 | 14511100 |  |  |  |  |  |  |  |
| T0W5N9 | Potassium transporter KefA | 14505500 |  |  |  |  |  |  |  |
| T0UZJ6 | 30S ribosomal protein S6 | 14442200 |  |  |  |  |  |  |  |
| T0W2N3 | Aspartate-semialdehyde dehydrogenase | 14427300 |  |  |  |  |  |  |  |
| T0UHW5 | Formate acetyltransferase | 14328700 |  |  |  |  |  |  |  |
| T0UMS7 | Alpha-L-Rha alpha-1,3-L-rhamnosyltransferase | 14197150 |  |  |  |  |  |  |  |
| T0VZ90 | 4-hydroxy-tetrahydrodipicolinate reductase | 14102950 |  |  |  |  |  |  |  |
| T0UG73 | Oligopeptidase PepB | 14027800 |  |  |  |  |  |  |  |
| T0W5Y3 | Elongation factor 4 | 14004200 |  |  |  |  |  |  |  |
| T0UTQ4;T0UZQ2;T0UFZ4 | Uncharacterized protein | 13862500 |  |  |  |  |  |  |  |
| T0UQC5 | [head protein]Uncharacterized protein | 13858750 |  |  |  |  |  |  |  |
| T0ULA9 | Cell division protein | 13733360 |  |  |  |  |  |  |  |
| T0VXT2 | Aminopeptidase | 13687100 |  |  |  |  |  |  |  |
| T0VYU8 | Hydrolase | 13578650 |  |  |  |  |  |  |  |
| T0ULB3 | CoA-disulfide reductase | 13486500 |  |  |  |  |  |  |  |
| T0UI77 | Uncharacterized protein | 13285065 |  |  |  |  |  |  |  |
| T0VLT1 | Peptide ABC transporter permease | 13281650 |  |  |  |  |  |  |  |
| T0UKF0 | Membrane protein | 13240150 |  |  |  |  |  |  |  |
| T0VWB0 | Lipoprotein | 13159050 |  |  |  |  |  |  |  |
| T0UW58 | 6-phosphogluconolactonase | 13157350 |  |  |  |  |  |  |  |
| T0V032;T0UZH9;T0UHA4 | Uncharacterized protein (Fragment) | 13122500 |  |  |  |  |  |  |  |
| T0UI65 | 6-phosphogluconate dehydrogenase, decarboxylating | 13058900 |  |  |  |  |  |  |  |
| T0UKJ4 | ArsR family transcriptional regulator | 12857850 |  |  |  |  |  |  |  |
| T0W1R6 | Adapter protein mecA | 12854250 |  |  |  |  |  |  |  |
| T0VWE3 | Primosomal protein DnaI | 12778850 |  |  |  |  |  |  |  |
| T0W005 | Lipoprotein | 12773200 |  |  |  |  |  |  |  |
| T0VTT1 | Rrf2 family transcriptional regulator | 12718300 |  |  |  |  |  |  |  |
| T0UVC6 | Peptidoglycan hydrolase | 12538700 |  |  |  |  |  |  |  |
| T0W8Z8 | DNA polymerase I | 12528550 |  |  |  |  |  |  |  |
| T0UC47 | Phosphomannomutase | 12492000 |  |  |  |  |  |  |  |
| T0UL74 | Phage infection protein | 12448850 |  |  |  |  |  |  |  |
| T0UFQ7 | Methylase | 12421650 |  |  |  |  |  |  |  |
| T0UYT3 | 50S ribosomal protein L11 | 12411650 |  |  |  |  |  |  |  |
| T0USS3 | Cell division protein DivIB | 12372150 |  |  |  |  |  |  |  |
| T0VZ88 | GDSL family lipase | 12309650 |  |  |  |  |  |  |  |
| T0UIR9 | GTPase | 12288050 |  |  |  |  |  |  |  |
| T0VSM1 | 3-oxoacyl-[acyl-carrier-protein] synthase 2 | 12272300 |  |  |  |  |  |  |  |
| T0VTY0 | Ferrous iron transport protein B | 12224300 |  |  |  |  |  |  |  |
| T0W1J5 | Uncharacterized protein | 12198560 |  |  |  |  |  |  |  |
| T0W6D8 | DNA gyrase subunit B | 12172900 |  |  |  |  |  |  |  |
| T0UPC0 | Glycine/betaine ABC transporter permease | 12165950 |  |  |  |  |  |  |  |
| T0UBV1 | Uncharacterized protein | 12035550 |  |  |  |  |  |  |  |
| T0UVA9 | Acetolactate synthase | 12028200 |  |  |  |  |  |  |  |
| T0VZ46 | Alpha/beta hydrolase | 11859750 |  |  |  |  |  |  |  |
| T0W5Q9 | Uncharacterized protein | 11641600 |  |  |  |  |  |  |  |
| T0W8N0 | Glucokinase | 11541000 |  |  |  |  |  |  |  |
| T0UQ40 | Peptidase M22 | 11495900 |  |  |  |  |  |  |  |
| T0UUW6 | Uncharacterized protein | 11495650 |  |  |  |  |  |  |  |
| T0VXV5 | Uncharacterized protein | 11395550 |  |  |  |  |  |  |  |
| T0UHG9 | Uncharacterized protein | 11365950 |  |  |  |  |  |  |  |
| T0USG4 | Aspartate carbamoyltransferase | 11359190 |  |  |  |  |  |  |  |
| T0V5F4 | ATP-dependent DNA helicase RecG | 11255400 |  |  |  |  |  |  |  |
| T0UF21 | 3-oxoacyl-[acyl-carrier-protein] reductase | 11202000 |  |  |  |  |  |  |  |
| T0W5B0 | Membrane protein | 11198250 |  |  |  |  |  |  |  |
| T0VWF5 | 2-succinyl-5-enolpyruvyl-6-hydroxy-3-cyclohexene-1-carboxylate synthase | 11192370 |  |  |  |  |  |  |  |
| T0W376 | Stress response regulator Gls24 | 11182700 |  |  |  |  |  |  |  |
| T0UKR8 | Cell division protein FtsJ | 11156050 |  |  |  |  |  |  |  |
| T0US64 | Phosphoglycerate mutase | 11135400 |  |  |  |  |  |  |  |
| T0UPU3 | GTP pyrophosphokinase | 10999050 |  |  |  |  |  |  |  |
| T0W2W7 | Thioredoxin reductase | 10961000 |  |  |  |  |  |  |  |
| T0UEG8 | Cytochrome D ubiquinol oxidase subunit I | 10955950 |  |  |  |  |  |  |  |
| T0UFC5 | Uncharacterized protein | 10931100 |  |  |  |  |  |  |  |
| T0W6H3 | Stress-responsive transcriptional regulator, PspC family | 10930250 |  |  |  |  |  |  |  |
| T0WFB5 | Ribose-5-phosphate isomerase A | 10757250 |  |  |  |  |  |  |  |
| T0VWG4 | Anaerobic ribonucleoside-triphosphate reductase-activating protein | 10693900 |  |  |  |  |  |  |  |
| T0W2I0 | UDP-N-acetylmuramyl peptide synthase | 10691200 |  |  |  |  |  |  |  |
| T0U9L4;T0VTG3 | RepB family protein (Fragment) T0VTG3; RepB family protein | 10658350 |  |  |  |  |  |  |  |
| T0UIW6 | Alanine acetyltransferase | 10607810 |  |  |  |  |  |  |  |
| T0UP71 | Lipoate--protein ligase | 10591850 |  |  |  |  |  |  |  |
| T0URJ1 | Polyribonucleotide nucleotidyltransferase | 10423650 |  |  |  |  |  |  |  |
| T0VR80 | Pantothenate kinase | 10418480 |  |  |  |  |  |  |  |
| T0W2W5 | S-adenosylmethionine:tRNA ribosyltransferase-isomerase | 10381965 |  |  |  |  |  |  |  |
| T0UF18 | Acetyl-coenzyme A carboxylase carboxyl transferase subunit beta | 10332300 |  |  |  |  |  |  |  |
| T0VNX4 | 23S rRNA methyltransferase | 10331150 |  |  |  |  |  |  |  |
| T0W5N8 | S1 RNA-binding protein | 10307850 |  |  |  |  |  |  |  |
| T0UI90 | Mevalonate kinase | 10223700 |  |  |  |  |  |  |  |
| T0VVE6 | Tryptophan--tRNA ligase | 10221900 |  |  |  |  |  |  |  |
| T0VZU8 | Alkyl hydroperoxide reductase C | 10196950 |  |  |  |  |  |  |  |
| T0UIU9 | Uncharacterized protein | 10173350 |  |  |  |  |  |  |  |
| T0UZI0 | Probable membrane transporter protein | 10169800 |  |  |  |  |  |  |  |
| T0UKV1 | Phosphate transport system permease protein | 10145250 |  |  |  |  |  |  |  |
| T0ULN1 | Phosphoribosylglycinamide formyltransferase | 10121275 |  |  |  |  |  |  |  |
| T0VY24 | Uncharacterized protein | 10006050 |  |  |  |  |  |  |  |
| T0UPV3 | Glutamate racemase | 10005750 |  |  |  |  |  |  |  |
| T0VSN0 | Transcription termination/antitermination protein NusA | 9998000 |  |  |  |  |  |  |  |
| T0UQ50 | Multidrug ABC transporter permease | 9993800 |  |  |  |  |  |  |  |
| T0VYX6 | Replication protein | 9858650 |  |  |  |  |  |  |  |
| T0W1Z8 | Phospho-N-acetylmuramoyl-pentapeptide-transferase | 9794685 |  |  |  |  |  |  |  |
| T0UQA7 | SAM-dependent methyltransferase | 9775700 |  |  |  |  |  |  |  |
| T0UPX3 | Lipoprotein | 9764490 |  |  |  |  |  |  |  |
| T0VWH7 | Energy-coupling factor transporter transmembrane protein EcfT | 9693750 |  |  |  |  |  |  |  |
| T0UJ60 | GntR family transcriptional regulator | 9651050 |  |  |  |  |  |  |  |
| T0UH18 | Uncharacterized protein | 9620100 |  |  |  |  |  |  |  |
| T0VWL2 | Peptidase_M22 domain-containing protein | 9581250 |  |  |  |  |  |  |  |
| T0W909 | Phosphatidate cytidylyltransferase | 9566500 |  |  |  |  |  |  |  |
| T0UIV6 | Multidrug ABC transporter ATP-binding protein | 9565950 |  |  |  |  |  |  |  |
| T0UQH7 | Phosphoglycerate mutase | 9510300 |  |  |  |  |  |  |  |
| T0VVM0 | Uncharacterized protein | 9454650 |  |  |  |  |  |  |  |
